# POU3F2 regulates canonical Wnt signalling via *SOX13* and *ADNP* to expand the neural progenitor population

**DOI:** 10.1093/brain/awaf221

**Published:** 2025-06-11

**Authors:** Courtney R Benoit, Lilia B Sattler, Aimee J Aylward, Olivia Pembridge, Bella Kim, Christina R Muratore, Meichen Liao, Amy He, Nancy Ashour, Seeley B Fancher, Alexandra M Lish, Richard V Pearse, Joseph D Buxbaum, Tracy L Young-Pearse

**Affiliations:** Ann Romney Center for Neurologic Diseases, Department of Neurology, Brigham and Women’s Hospital and Harvard Medical School, Boston, MA 02115, USA; Ann Romney Center for Neurologic Diseases, Department of Neurology, Brigham and Women’s Hospital and Harvard Medical School, Boston, MA 02115, USA; Ann Romney Center for Neurologic Diseases, Department of Neurology, Brigham and Women’s Hospital and Harvard Medical School, Boston, MA 02115, USA; Ann Romney Center for Neurologic Diseases, Department of Neurology, Brigham and Women’s Hospital and Harvard Medical School, Boston, MA 02115, USA; Ann Romney Center for Neurologic Diseases, Department of Neurology, Brigham and Women’s Hospital and Harvard Medical School, Boston, MA 02115, USA; Ann Romney Center for Neurologic Diseases, Department of Neurology, Brigham and Women’s Hospital and Harvard Medical School, Boston, MA 02115, USA; Ann Romney Center for Neurologic Diseases, Department of Neurology, Brigham and Women’s Hospital and Harvard Medical School, Boston, MA 02115, USA; Ann Romney Center for Neurologic Diseases, Department of Neurology, Brigham and Women’s Hospital and Harvard Medical School, Boston, MA 02115, USA; Ann Romney Center for Neurologic Diseases, Department of Neurology, Brigham and Women’s Hospital and Harvard Medical School, Boston, MA 02115, USA; Ann Romney Center for Neurologic Diseases, Department of Neurology, Brigham and Women’s Hospital and Harvard Medical School, Boston, MA 02115, USA; Ann Romney Center for Neurologic Diseases, Department of Neurology, Brigham and Women’s Hospital and Harvard Medical School, Boston, MA 02115, USA; Ann Romney Center for Neurologic Diseases, Department of Neurology, Brigham and Women’s Hospital and Harvard Medical School, Boston, MA 02115, USA; Department of Psychiatry, Icahn School of Medicine at Mount Sinai, New York, NY 10029, USA; Ann Romney Center for Neurologic Diseases, Department of Neurology, Brigham and Women’s Hospital and Harvard Medical School, Boston, MA 02115, USA

**Keywords:** canonical Wnt signalling, induced pluripotent stem cells (iPSCs), neural progenitor cells (NPCs), CRISPR/Cas9, neurodevelopmental disorders, autism spectrum disorder (ASD)

## Abstract

Loss-of-function mutations in the transcription factor *POU3F2* have been identified in individuals with neurodevelopmental disorders. To elucidate the mechanistic role of POU3F2 in human neurodevelopment, we induced *POU3F2* disruption in human neural progenitor cells (NPCs). Mutation of *POU3F2* in NPCs causes reduced baseline canonical Wnt signalling and decreased proliferation, resulting in premature specification of radial glia. Additionally, *POU3F2* levels across genetically diverse NPCs significantly associate positively with baseline canonical Wnt signalling and negatively with markers of radial glia specification.

Through a series of unbiased analyses, we show that SRY-box transcription factor 13 (SOX13) and activity dependent neuroprotector homeobox (ADNP) are transcriptional targets of POU3F2 which mediate POU3F2's effects on Wnt signalling in human NPCs. Finally, we describe five individuals with autism spectrum disorder that harbour loss-of-function mutations in *POU3F2*, enhancing the genetic evidence for its critical role in human neurodevelopment. Together, these studies define POU3F2 as an activator of canonical Wnt signalling and mechanistically link two high-confidence autism genes, *ADNP* and *POU3F2*, in the regulation of neurodevelopment.

## Teaser

Using cellular models of human brain development, Benoit *et al.* investigate the role of POU3F2, a transcription factor that has been implicated in neurodevelopmental disorders. They conclude that POU3F2 acts to enhance canonical Wnt signalling, a pathway central to neurodevelopment.

## Introduction

Proper mammalian neurodevelopment relies on tightly regulated gene networks controlled by neural transcription factors. Disruptions in this process are linked to disorders such as developmental delay, intellectual disability, autism spectrum disorder (ASD) and neuropsychiatric conditions.^1-4^ Precise modulation of the Wnt signalling pathway is critical for proper neurodevelopment,^5-7^ and its dysregulation is implicated in a range of neurodevelopmental disorders.^8-11^ Lithium, a treatment for bipolar disorder, inhibits glycogen synthase kinase 3β (GSK3β), a key inhibitor of canonical Wnt signalling.^12^ Additionally, altered pathway components consistent with reduced canonical (β-catenin-dependent) and increased non-canonical (β-catenin-independent) Wnt signalling have been observed in plasma from individuals with schizophrenia and bipolar disorder.^13^ Similarly, induced pluripotent stem cell (iPSC)-derived neurons from individuals with schizophrenia show aberrant Wnt-related gene expression.^14^ Our group has also linked disruption of *DISC1*, a gene implicated in familial neuropsychiatric disease, to Wnt signalling abnormalities in human neural cells.^15,16^ Further, *de novo* mutations in canonical Wnt modulators such as *ADNP, CTNNB1* and *TCF7L2* have been associated with ASD.^17,18^

*POU3F2* encodes a neural-specific Pit-Oct-Unc (POU) class III transcription factor^19^ implicated in neurodevelopmental disorders. Overlapping 6q16.1 heterozygous deletions encompassing *POU3F2* were identified in 10 individuals from 6 families with shared features including developmental delay and intellectual disability.^20^ Further, a *de novo* missense mutation in the POU-specific domain^21^ of *POU3F2* (c.812A > T, p.Glu271Val) was found in an individual with developmental delay.^22^ A recent study reported an additional 11 individuals with monoallelic *POU3F2* variants, 10 of whom presented with intellectual disability.^23^ Genetic studies have also linked *POU3F2* variants to bipolar disorder,^24-26^ schizophrenia^27^ and ASD.^28,29^ In a transcriptomic study, POU3F2 targets were found to be overexpressed in the cortex of individuals with neuropsychiatric disease.^30^ Additionally, POU3F2 was identified as a regulator of a schizophrenia-associated co-expression module^31^ and found to affect transcription of 42 schizophrenia-linked genes, including *TRIM8.*^32^

While these studies underscore the importance of POU3F2 in neurodevelopment, little is known about the mechanisms underlying its connection to human disorders. In mice, *Pou3f2* is critical for hypothalamic neurogenesis, and ubiquitous loss of *Pou3f2* results in postnatal lethality.^33,34^ Complete loss of neurogenesis is restricted to the hypothalamus, likely as a result of partial redundancy with its paralogue *Pou3f3.*^35^ Conversely, cortical overexpression of *Pou3f2* increases upper-layer neurons and decreases deep-layer neurons.^36^ Double *Pou3f2*/*Pou3f3* knockout mice exhibit severe cortical defects, including layer inversion and reduced progenitor proliferation.^37^ Cortex-specific *Pou3f2/Pou3f3* knockouts further revealed a shift from indirect to direct neurogenesis and dysregulation of microcephaly-associated genes.^38^ Deletion of the mammalian-specific amino acid repeat regions in *Pou3f2* was found to affect maternal behaviour,^39,40^ social activity^41^ and adult hippocampal neurogenesis^42^ in mice. In non-human primates, CRISPR/Cas9-mediated *POU3F2* knockout reduced vertical divisions of neural progenitors and induced precocious neurodifferentiation, phenotypes which were replicated in human organoids.^43^ Further, two independent studies using human models have shown that short hairpin RNA (shRNA)-mediated partial knockdown of *POU3F2* impairs neuronal differentiation.^32,44^

In this study, we investigated how POU3F2 affects early neurodevelopment using human cellular models. We generated *POU3F2* loss-of-function mutations in human iPSCs and differentiated these into neural progenitor cells (NPCs). Unbiased transcriptomic and proteomic analyses revealed dysregulation of Wnt signalling components in *POU3F2* mutant (POU3F2^MUT^) NPCs, accompanied by decreased baseline canonical Wnt signalling and reduced proliferation. Single-cell analysis showed a shift in NPC subtypes in POU3F2^MUT^ NPCs favouring increased radial glia specification, a phenotype that we validated in POU3F2^MUT^ cerebral organoids. Using a large cohort of genetically diverse lines, we found that natural variation in *POU3F2* levels positively associates with baseline canonical Wnt signalling and negatively associates with radial glia specification, mirroring loss-of-function phenotypes. We then used unbiased approaches to identify candidate mechanistic effectors, and found that POU3F2 directly regulates *SOX13* and *ADNP*, which together mediate POU3F2's effects on Wnt signalling. ADNP was also found to interact with POU3F2, further supporting a mechanistic link between these proteins. Finally, we show that genes dysregulated in ASD are enriched in multiple *POU3F2*-related datasets generated in this study, and we describe five previously unreported individuals with ASD that harbour deletions or mutations in *POU3F2*. These data support the hypothesis that POU3F2 activates canonical Wnt signalling to regulate NPC expansion and implicate *POU3F2* disruption in ASD pathogenesis.

## Materials and methods

### Generation of CRISPR/Cas9-edited iPSCs

CRISPR/Cas9 mutagenesis of *POU3F2* was performed in three iPSC lines: YZ1, derived from fetal fibroblasts,^45^ and BR33 and BR24, derived from cognitively unimpaired individuals from the Religious Orders Study and Rush Memory and Aging Project (ROSMAP) cohorts.^46-49^ All iPSCs underwent extensive quality control, including sterility, mycoplasma and karyotype testing. A single guide RNA (sgRNA) was designed using the Zhang Lab CRISPR Design Tool (crispr.mit.edu) to target the POU-specific domain of *POU3F2,* cloned into a Cas9-expressing vector (Addgene) and sequence verified. iPSCs were electroporated with the sgRNA plasmid, monoclonally selected and Sanger sequenced to identify mutations. For each genetic background, one wild-type and two mutant clones were selected for downstream analyses. Colour-coding is used throughout figures to indicate genetic background (green = YZ1, blue = BR33, pink = BR24), with corresponding details in Supplementary Table 1. *SOX13* wild-type and mutant clones were generated in BR33 as described using an sgRNA targeting a constitutively expressed exon.

### Differentiation of iPSC-derived NPCs

NPCs were differentiated using a dual-SMAD (Suppressor of Mothers Against Decapentaplegic) inhibition protocol via an embryoid body (EB) intermediate.^50^ Confluent iPSCs were dissociated with collagenase IV, transferred to non-adherent plates and fed every other day with Dulbecco's Modified Eagle's Medium (DMEM)-F12/N2/B27-RA media containing 100 nM LDN193189 (Stemgent) and 10 μM SB431542 (STEMCELL). On Day 7 (D7), EBs were resuspended in the above media supplemented with 1 μg/ml laminin (Gibco), plated on poly-ornithine/laminin (POL)-coated plates and fed every other day. On D14, neural rosettes were isolated using the STEMdiff^TM^ neural rosette selection reagent (STEMCELL) to enrich cultures for NPCs and to eliminate non-neural cells. Rosettes were resuspended in NPC media [DMEM-F12/N2/B27-RA with 1 μg/ml laminin and 20 ng/ml fibroblast growth factor 2 (FGF2) (Peprotech)], replated on POL-coated plates and fed every other day. After D21, NPCs were expanded on growth factor-reduced (GFR) Matrigel (Corning) at high density for up to three passages. NPC composition was confirmed via immunostaining for cell fate markers (NPC: PAX6/Nestin; neuron: MAP2; astrocyte: CD44).

### Differentiation of iPSC-derived cerebral organoids

YZ1 and BR24 cerebral organoids were generated as described^51^ with minor modifications.^16^ BR33 cerebral organoids were generated as described.^52^ Organoids were fixed in 4% paraformaldehyde, cryosectioned (15 μm; YZ1/BR33 = D60, BR24 = D83), and blocked with 3% bovine serum albumin (BSA)/0.1% Triton-X-100 (Sigma) for 1 h at room temperature. Sections were incubated overnight at 4°C with primary antibodies diluted in 1% BSA: ANXA1 (Abcam, Cat. No. 214486, 1:100), SOX2 (R&D Systems, Cat. No. AF2018, 1:500), Nestin (Novus Biologicals, Cat. No. MAB1259, 1:500), VIM (R&D Systems, Cat. No. MAB2105, 1:100), BLBP (R&D Systems, Cat. No. AF3166, 1:100). Secondary antibodies (1:2000 dilution) were applied for 1 h, followed by 4',6-diamidino-2-phenylindole (DAPI) staining (1:1000 dilution) for 10 min, and VECTASHIELD mounting (Vector Laboratories). Imaging was performed on Zeiss LSM710 or LSM880 confocal microscopes (NeuroTechnology Studio, BWH), and all image quantification was conducted blinded to *POU3F2* genotype.

### Differentiation of iPSC-derived brain cell types

*Ngn2-*induced neurons (iNs) were differentiated as described^53^ with minor modifications.^47,54^ Astrocytes (ebAstros) were differentiated from NPCs as described.^49^ Induced microglia (iMGLs) were differentiated following previously published papers^55,56^ with minor modifications.^47,57^

### Immunocytochemistry

Cells were fixed in 4% paraformaldehyde for 15 min at room temperature then blocked in 2% donkey serum (Jackson ImmunoResearch) and 0.2% Triton-X-100 (Sigma) for 1 h at room temperature. Cells were incubated with primary antibody diluted in blocking buffer overnight at 4°C: POU3F2 (CST, Cat. No. 12137S, 1:100), Nestin (Novus Biologicals, Cat. No. MAB1259, 1:500), PAX6 (Biolegend, Cat. No. 901301, 1:400), SOX2 (R&D Systems, Cat. No. AF2018, 1:500), MAP2 (Abcam, Cat. No. ab5392, 1:500), CD44 (Abcam, Cat. No. ab254530, 1:500), ANXA1 (Abcam, Cat. No. 214486, 1:100), BLBP (R&D Systems, Cat. No. AF3166, 1:100), ADNP (Abcam, Cat. No. ab300114, 1:100), SOX13 (Proteintech, Cat. No. 18902-1, 1:100), β-catenin (CST, Cat. No. 8480S, 1:100). Secondary antibodies (1:2000 dilution) were applied for 1 h, followed by DAPI staining (1:1000 dilution) for 10 min. Imaging was performed using a Zeiss LSM710 confocal microscope or GE INCELL Analyzer 2200 (NeuroTechnology Studio, BWH). When applicable, per cent positivity was quantified using CellProfiler.^58^

### Western blotting

Cells were lysed in radioimmunoprecipitation assay (RIPA) buffer (ThermoFisher) supplemented with cOmplete^TM^ Mini protease inhibitor and PhosSTOP^TM^ phosphatase inhibitor (Millipore Sigma) for 30 min on ice. Lysates were centrifuged at 13 000*g* for 10 min at 4°C and supernatants were collected. Protein concentration was measured using the Pierce BCA Assay (ThermoFisher). Lysates were prepared with 4× LI-COR loading buffer (ThermoFisher) and 2.5% β-mercaptoethanol and heated at 95°C for 10 min. Proteins were resolved using NuPAGE^TM^ 4%–12% Bis(2-hydroxyethyl)imino-tris(hydroxymethyl)methane (Bis-Tris) gels (ThermoFisher) and NuPAGE^TM^ 1x 3-(N-morpholino)propanesulfonic acid-sodium dodecyl sulfate (MOPS-SDS) buffer (ThermoFisher) at 100 V for 15 min, followed by 150 V for 75 min. SeeBlue Plus2 (ThermoFisher) standard was used to assess molecular weight. Proteins were transferred to nitrocellulose membranes in 20% methanol Tris-glycine transfer buffer at 400 mA for 2 h, then blocked with Odyssey blocking buffer (LI-COR) for 1 h at room temperature. Primary antibodies diluted in blocking buffer were applied overnight at 4°C: POU3F2 (CST, Cat. No. 12137S, 1:1000), GAPDH (Proteintech, Cat. No. 60004-1-Ig, 1:10 000), β-catenin (CST, Cat. No. 8480S, 1:1000), Nestin (Novus Biologicals, Cat. No. MAB1259, 1:1000), TUJ1 (Novus Biologicals, Cat. No. NB100-1612, 1:1000), POU3F2 (Santa Cruz, Cat. No. sc-393324, 1:1000), HA-Tag (CST, Cat. No. C29F4, 1:500), ADNP (Abcam, Cat. No. ab300114, 1:500), SOX13 (Proteintech, Cat. No. 18902-1, 1:1000). Blots were incubated with LI-COR secondary antibodies (1:10 000 dilution) for 1 h at room temperature. Blots were imaged on a LI-COR Odyssey machine and quantified with ImageStudio software.

### Bulk RNA-sequencing

RNA was extracted using the PureLink RNA Mini Kit (ThermoFisher), and quality was assessed with Tapestation RNA ScreenTape (Agilent). Samples were submitted to Genewiz (Azenta) for library preparation and sequencing. Fastq file quality was assessed with fastqc.^59^ Reads were pseudo-aligned with Kallisto.^60^ Counts were normalized, filtered for low-abundance transcripts and batch effects regressed using ComBat (sva).^61^ Principal component analysis (PCA) was performed on the batch-corrected, log-transformed matrix using prcomp.^62^ Differential expression was calculated in Sleuth.^63^ Pathway enrichment was assessed against the Reactome database using clusterProfiler^64^ and enrichment dot-plots were generated using enrichplot.^65^ Heatmaps were created using pheatmap^66^ with colour palettes from Seurat.^67^

### Proteomics via tandem mass tag-mass spectrometry

NPCs were plated in 6-well plates (1 million cells/well) and grown to confluency. Cells were lysed in 8 M urea and sent to the Emory Integrated Proteomics Core for peptide identification. Samples underwent tandem mass tag (TMT) labelling and were analysed via liquid chromatography-mass spectrometry (LC-MS)/MS. Abundance data were log_2_ transformed, and batch effects were regressed using ComBat.^61^ Differential abundance was calculated using Differential Enrichment analysis of Proteomics data (DEP).^68^ Gene set enrichment (Broad Hallmark) was performed using fgsea^69^ and results were plotted using ggplot2.

### SUPERTOPFLASH luciferase Wnt reporter assay

Mouse fibroblasts untransfected (Control; ATCC) or stably transfected with a Wnt3a-overexpression vector (Wnt3a; ATCC) were used to produce control or Wnt3a-conditioned media. Fibroblasts were maintained in DMEM containing 10% fetal bovine serum (FBS), and media was collected per American Type Culture Collection (ATCC) guidelines, aliquoted and stored at −80°C. For the SUPERTOPFLASH assay, NPCs were nucleofected with two reporter plasmids: pRL-CMV (*Renilla* luciferase under CMV promoter), and pGL4.49(*luc2P*/TCF-LEF RE/Hygro) (firefly luciferase under minimal promoter with a T-cell factor/lymphoid enhancer factor (TCF/LEF) response element) (Promega). Nucleofection was performed using the Amaxa P3 Nucleofector Kit (Lonza) at a ratio of 1.8 μg pGL4.49(*luc2P*/TCF-LEF RE/Hygro) and 0.2 μg pRL-CMV per 1 million cells. Nucleofected cells were plated in white-walled 96-well plates (Corning) at 100 k cells/well. After 24 h, media was replaced with control or Wnt3a-conditioned media. Following another 24 h, luciferase activity was measured using the Dual-Luciferase Reporter Assay (Promega) on a spectrophotometer with 5-s signal integration. Baseline Wnt signalling was calculated as Firefly/*Renilla* in control-treated samples, while activated canonical Wnt signalling was calculated by dividing this ratio in Wnt3a-treated samples by the ratio in control-treated samples. For overexpression experiments, the assay was performed as described with the addition of 1 μg pmaxGFP (Lonza), 1 μg human FLAG-tagged *SOX13* vector (Origene, Cat. No. RC210697) or 1 μg human HA-tagged *ADNP* vector (VectorBuilder, pRP(Exp)-CAG > HA/hADNP) per 1 million cells.

### Proliferation assay

NPCs plated at 30 k cells/well in 96-well plates were treated with 10 μM 5-ethynyl-2'-deoxyuridine (EdU) (Invitrogen) for 6 h, then fixed with 4% paraformaldehyde and permeabilized with 0.5% Triton X-100. EdU detection followed the manufacturer's protocol, and nuclei were counterstained with Hoechst33342. Images were acquired using a GE INCELL Analyzer 2200 (NeuroTechnology Studio, BWH). Per cent positivity was calculated using CellProfiler.^58^

### Gene set enrichment analyses

Gene set enrichment analyses (GSEA) were performed using fgsea.^69^ Only genes expressed above a threshold (count >2 in ≥30% of samples) were included as the baseline set. Custom .gmt files were imported using fgsea. For Wnt signalling targets, differential expression results from Wnt3a- versus control-treated NPCs were ranked (−log_10_ adjusted *P*-value * log_2_ fold change), and the top 100 upregulated and downregulated unique genes were used as the ‘Wnt activated’ and ‘Wnt repressed’ gene sets. G2/M progenitor and radial glial markers were defined in NPCs that clustered with the corresponding subtypes in human fetal brain using the Seurat function ‘FindAllMarkers’.^67^ For POU3F2-regulation gene sets, genes and proteins were defined as upregulated or downregulated in POU3F2^MUT^ NPCs with adjusted *P*-value <0.05.

### Single-nucleus RNA-seq of iNs, ebAstros and iMGLs

For iNs and ebAstros, cells were washed in 0.04% BSA and lysed on ice for 15 min (10 mM Tris, 0.49% 3-[(3-cholamidopropyl)dimethylammonio]-1-propanesulphonate (CHAPS), 0.1% BSA, 21 mM MgCl_2_, 1 mM CaCl_2_, 146 mM NaCl). Nuclei were pelleted by centrifugation (5 min, 500*g*) and resuspended in 1% BSA. For iMGLs, cells were first pelleted, then lysed and resuspended as above. Filtered single-nuclei suspensions were loaded into a 10× Chromium Controller (NeuroTechnology Studio, BWH). Libraries were prepared per manufacturer's instructions, assessed for quality with a TapeStation and sequenced at the Broad Institute. Reads were processed with CellRanger^70^ and imported into Seurat.^67^ Nuclei with >10% mitochondrial reads or with <200 or >6000 detected features were excluded.

### Single-cell RNA-seq of POU3F2^WT^ and POU3F2^MUT^ NPCs

NPCs were dissociated with 1:1 accutase:PBS (Invitrogen) and filtered for debris using 30 μm filters (Sysmex). Cells were encapsulated using a 10x Chromium Controller (NeuroTechnology Studio, BWH), followed by library preparation and sequencing (Harvard Biopolymers Facility). Reads were demultiplexed,^71^ processed with CellRanger^70^ and imported into Seurat.^67^ Cells with >10% mitochondrial reads or with <200 or >6000 detected features were excluded. Harmony^72^ was used to regress sex effects prior to integration. POU3F2^WT^ and POU3F2^MUT^ NPCs were integrated with: (i) snRNA-seq data from iPSC-derived brain cells; or (ii) scRNA-seq data from a Carnegie Stage 14 (CS14) fetal brain.^73^ For CS14 fetal brain integration, cells were filtered in Seurat as above. Harmony^72^ was performed to regress the effect of origin (i.e. ‘brain’ versus ‘NPC’) and uniform manifold approximation and projection (UMAP)^74^ was used to visualize integration. Marker genes were identified in CS14 fetal brain using the ‘FindAllMarkers’ function^67^ and clusters were annotated based on marker expression. Pseudotime analysis was performed using monocle3^75-77^ and plotted with ggridges,^78^ with t = 0 defined as the node farthest from the immature neuron cluster (highest value on UMAP_2). Chi-square tests were performed to compare cell type proportions between genotypes, using wild-type ratios as expected values.

### Gene set variation analyses

Participant-derived NPCs were differentiated and plated in parallel for protein extraction and RNA-seq. Gene set variation analysis (GSVA) was applied to bulk RNA-seq data using the previously described ‘Wnt activated’ gene set. Scores were range-scaled (0 = lowest, 1 = highest), and Pearson correlation with *POU3F2* log_2_ expression was calculated.

### Cleavage under targets and release using nuclease

NPCs were dissociated with 1:1 accutase:PBS (Invitrogen). Cleavage under targets and release using nuclease (CUT&RUN) was performed per manufacturer's instructions with minor modifications (CST). For each reaction, 100 k cells were collected, washed in buffer containing protease inhibitors and spermidine, bound to Concanavalin A beads and incubated overnight at 4°C with primary antibody at 1:50 dilution (POU3F2: CST, Cat. No. 12137S; H3K4me3: CST, Cat. No. 9751). Input samples were treated with proteinase K and RNase A, then sonicated (Bioruptor Plus; 15 cycles, 30 s on/off, high intensity). Input lysates were clarified and stored at −20°C. CUT&RUN samples were permeabilized with digitonin, incubated with pAG-MNase and activated by CaCl_2_. Released DNA fragments were collected via centrifugation. Input and CUT&RUN samples were purified with spin columns and quantified with a Qubit double-stranded DNA (dsDNA) HS assay (ThermoFisher). Libraries were prepared (CST) and sequenced [2 × 75 base pairs (bp), Harvard Biopolymers Facility]. Read quality was assessed with FastQC,^59^ and reads were aligned to hg38 using bowtie2.^79^ Alignments were filtered with samtools^80^ to remove unmapped reads, duplicate reads and reads >300 bp. Peaks were called with Model-based Analysis of ChIP-Seq (MACS2)^81^ compared with inputs, and differential peaks were scored using DiffBind.^82^ Peaks were annotated and screened for motif enrichment using Hypergeometric Optimization of Motif EnRichment (HOMER).^83^

### Immunoprecipitation followed by mass spectrometry

NPCs were lysed in 1% 3-((3-cholamidopropyl)dimethylammonio)-2-hydroxy-1-propanesulfonate (CHAPSO) buffer, and protein concentration was measured using the Pierce bicinchoninic acid (BCA) Assay (ThermoFisher). Lysates were pre-cleared with Protein G Dynabeads (Invitrogen). Primary antibodies were cross-linked to Dynabeads using bis(sulfosuccinimidyl)suberate (BS^3^) (ThermoFisher). Antibodies used for immunoprecipitation include: POU3F2 (CST, Cat. No. 12137S, 1:50) and immunoglobulin G (IgG) (ThermoFisher, Cat. No. 02-6102, 0.4 μg/reaction). Pre-cleared lysates were incubated with the antibody-bead complex overnight at 4°C. For western blot, beads were washed with CHAPSO buffer, proteins were eluted with 5% β-mercaptoethanol and samples were run on a NuPAGE^TM^ 4%–12% Bis-Tris gel (ThermoFisher). For mass spectrometry, beads were washed with CHAPSO buffer and flash-frozen at −80°C. Samples were sent to the Emory Integrated Proteomics Core for peptide identification. Enrichment analysis was performed in Perseus^84^ following core facility guidance.

### Data visualization

Schematics were generated in BioRender. Graphs and heatmaps were generated using Prism 9.0 for Mac (GraphPad) or R.^62^

### Statistical analyses

All statistical tests were performed in Prism 9.0 for Mac (GraphPad) or in R.^62^

## Results

### CRISPR/Cas9-mediated mutagenesis of *POU3F2* in human iPSCs

**Figure 1:**
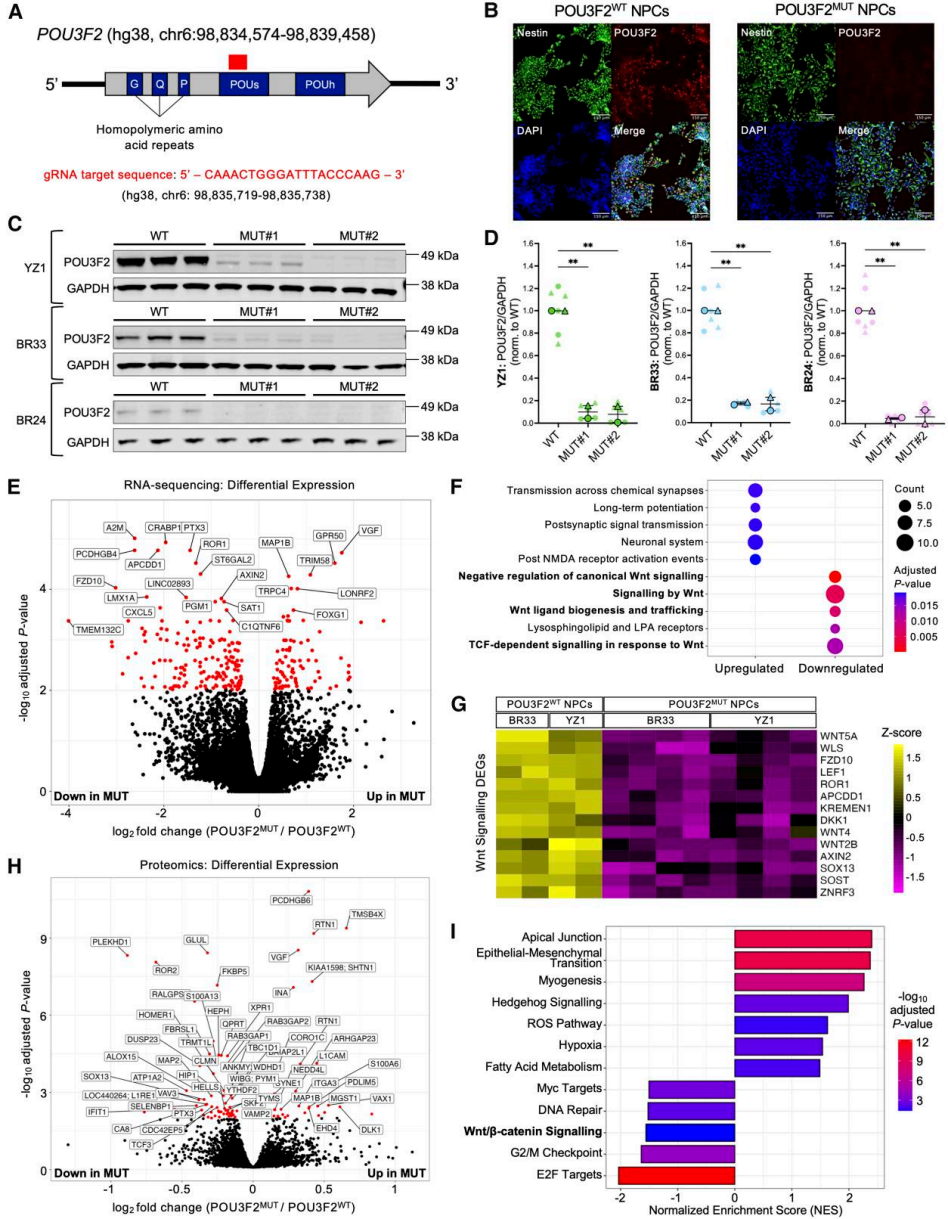
**Unbiased analyses of loss-of-function alleles of *POU3F2* in human NPCs implicate consequences on canonical Wnt signalling**. (**A**) Genomic structure of *POU3F2* with conserved domains labelled. Box indicates relative position of gRNA target sequence. POUs: POU-specific domain; POUh: POU homeodomain. (**B**) Immunocytochemistry of Nestin (NPC marker) and POU3F2 in POU3F2^WT^ and POU3F2^MUT^ NPCs. Scale bar = 150 μm. (**C**) Western blot analysis of POU3F2^WT^ and POU3F2^MUT^ NPCs, probing for POU3F2 and GAPDH. (**D**) Quantification of POU3F2 expression across POU3F2^WT^ and POU3F2^MUT^ NPCs, mean ± SEM (*n* = 2 differentiations). Mixed-effects model with *post hoc* Dunnett's test, **P < 0.01. (**E**) Volcano plot of differential gene expression results from bulk RNA-seq (MUT versus WT). Significance threshold = genes with adjusted *P*-value < 0.01 (*n* = 2 samples per line). (**F**) Gene ontology pathway enrichment results of differentially expressed genes in POU3F2^MUT^ NPCs, separated by directionality of log_2_ fold change. (**G**) Heat map of genes downregulated in POU3F2^MUT^ NPCs that are related to Wnt signalling. Values in heat map correspond to scaled expression values for each gene (row *Z*-score). (**H**) Volcano plot of differential expression results from proteomics (MUT versus WT). Significance threshold = proteins with adjusted *P*-value < 0.01. (**I**) Hallmark pathway gene set enrichment results from proteomics differential expression analysis. gRNA = guide RNA; MUT = mutant; NPC = neural progenitor cell; SEM = standard error of the mean; WT = wild-type.

To assess POU3F2's role in neurodevelopment, we performed CRISPR/Cas9 mutagenesis in three human iPSC lines (YZ1, BR33, BR24) with a guide RNA (gRNA) targeted to the POU-specific domain of *POU3F2* (Fig. 1A). From each parental line, we selected one wild-type (WT; POU3F2^WT^) and two mutant (MUT; POU3F2^MUT^) clones with compound heterozygous, protein-disrupting mutations (Supplementary Fig. 1A). POU3F2^WT^ and POU3F2^MUT^ iPSCs were differentiated into NPCs using dual-SMAD inhibition.^50^ Immunocytochemistry showed comparable Nestin expression across genotypes, while POU3F2 expression was restricted to POU3F2^WT^ NPCs (Fig. 1B). Western blot analysis confirmed marked reduction of POU3F2 protein in all mutant lines (Fig. 1C and D and Supplementary Fig. 1B and C). Lines used in each subsequent figure panel are indicated by symbol colour and further detailed in Supplementary Table 1.

### Unbiased analyses reveal disrupted Wnt signalling in POU3F2^MUT^ NPCs

POU3F2^WT^ and POU3F2^MUT^ NPCs were analysed by bulk RNA-seq to identify differentially expressed genes (Fig. 1E and Supplementary Tables 2 and 3). Reactome pathway^85^ enrichment analysis revealed upregulation of neuronal/synaptic pathways and downregulation of Wnt-related pathways in POU3F2^MUT^ NPCs (Fig. 1F). A heat map of canonical Wnt pathway genes showed clear distinction between *POU3F2* genotypes (Fig. 1G). Proteomic profiling via TMT-MS revealed differentially abundant proteins between POU3F2^WT^ and POU3F2^MUT^ NPCs (Fig. 1H and Supplementary Table 4). GSEA queried against Hallmark pathways^86,87^ (Broad Institute) corroborated transcriptomic findings, confirming downregulation of Wnt/β-catenin signalling proteins in POU3F2^MUT^ NPCs (Fig. 1I). Collectively, these unbiased analyses support a functional connection between POU3F2 and canonical Wnt signalling in human NPCs.

### POU3F2^MUT^ NPCs exhibit reduced canonical Wnt signalling and proliferation

**Figure 2:**
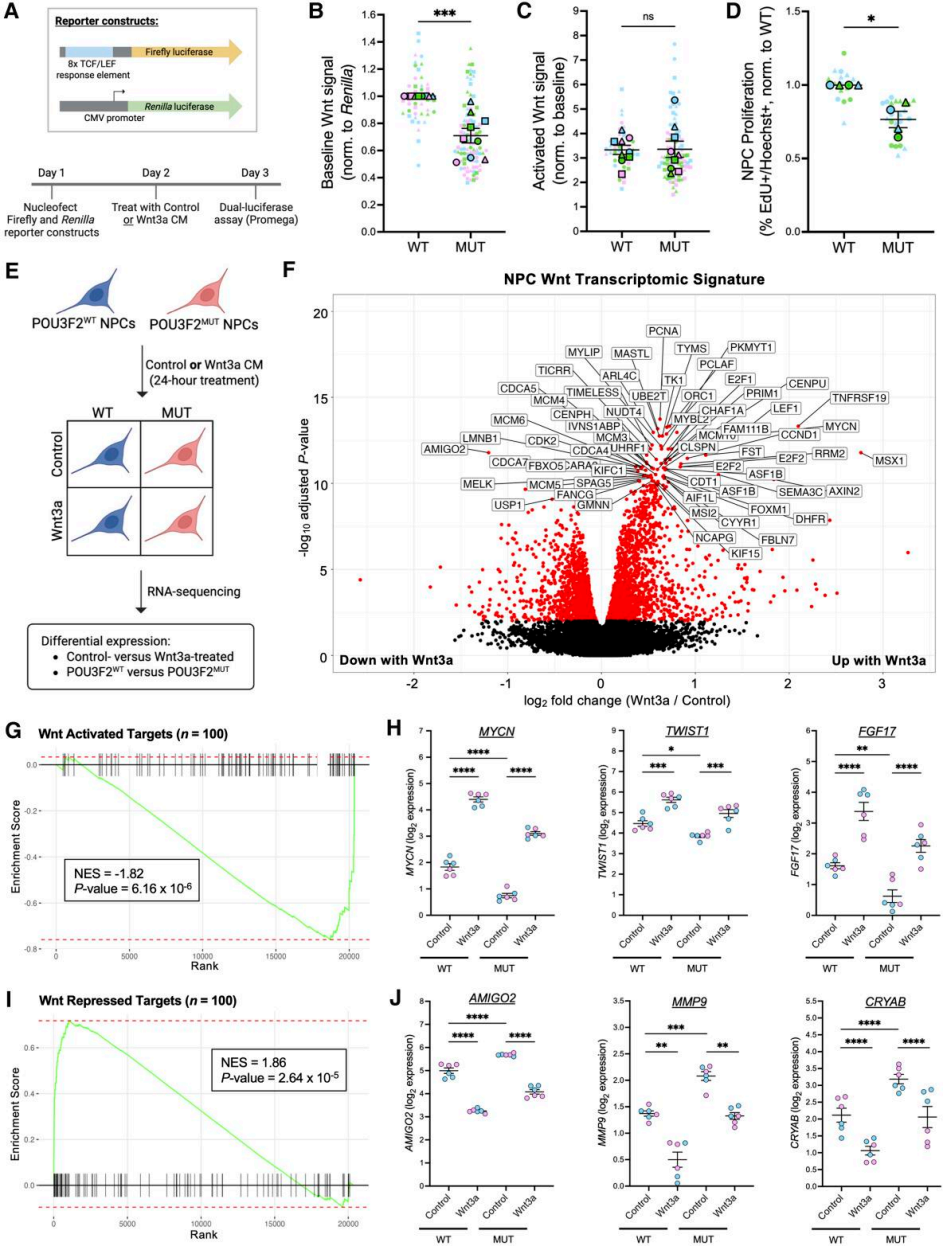
**POU3F2^MUT^ NPCs exhibit reduced canonical Wnt signalling and proliferation.** (**A**) Schematic of SUPERTOPFLASH reporter constructs and assay timeline. (**B**) Baseline canonical Wnt signalling, as measured by a SUPERTOPFLASH assay, in POU3F2^WT^ and POU3F2^MUT^ NPCs, mean ± SEM (*n* = 9 differentiations). Paired *t*-test, ***P < 0.001. (**C**) Activated canonical Wnt signalling, as measured by a SUPERTOPFLASH assay, in POU3F2^WT^ and POU3F2^MUT^ NPCs treated with Wnt3a-conditioned media, mean ± SEM (*n* = 9 differentiations). Paired *t*-test, ns = not significant. (**D**) EdU incorporation assay in POU3F2^WT^ and POU3F2^MUT^ NPCs, mean ± SEM (*n* = 4 differentiations). Paired *t*-test, *P < 0.05. (**E**) Schematic of experimental design for RNA-seq of POU3F2^WT^ and POU3F2^MUT^ NPCs treated with control or Wnt3a-conditioned media. (**F**) Volcano plot of differential gene expression results (Wnt3a treated versus control treated). Significance threshold = genes with adjusted *P*-value <0.01 (*n* = 3 samples per line for each condition). (**G**) GSEA of Wnt-activated targets (top 100, ranked by -log_10_ adjusted *P*-value * log_2_ fold change) in POU3F2^MUT^ versus POU3F2^WT^ differential expression. (**H**) Expression of leading-edge genes that are activated by canonical Wnt signalling, mean ± SEM. Mixed-effects model with *post hoc* Sidak's test, *P < 0.05, **P < 0.01, ***P < 0.001, ****P < 0.0001. (**I**) GSEA of Wnt-repressed targets (top 100, ranked by -log_10_ adjusted *P*-value * log_2_ fold change) in POU3F2^MUT^ versus POU3F2^WT^ differential expression. (**J**) Expression of leading-edge genes that are repressed by canonical Wnt signalling, mean ± SEM. Mixed-effects model with *post hoc* Sidak's test, **P < 0.01, ***P < 0.001, ****P < 0.0001. Schematics created in BioRender. Benoit, C. (2025) https://BioRender.com/5nz5qvx. EdU = 5-ethynyl-2'-deoxyuridine; GSEA = gene set enrichment analysis; MUT = mutant; NPC = neural progenitor cell; SEM = standard error of the mean; WT = wild-type.

To test whether the transcriptomic and proteomic changes reflect altered Wnt signalling, we performed the well-established SUPERTOPFLASH luciferase assay^88^ (Fig. 2A). Baseline canonical Wnt signalling was significantly reduced in POU3F2^MUT^ NPCs (Fig. 2B and Supplementary Fig. 1D). However, both genotypes showed comparable induction of Wnt activation following Wnt3a treatment (Fig. 2C), indicating intact responsiveness but impaired basal regulation. As Wnt signalling can impact mitotic cell proliferation, EdU incorporation assays were performed. POU3F2^MUT^ NPCs showed reduced basal proliferation compared with wild-type, which could be restored with Wnt3a treatment (Fig. 2D and Supplementary Fig. 1E and F). These findings suggest that POU3F2 regulates both baseline Wnt activity and NPC proliferation.

### Transcriptomic signatures of canonical Wnt activation and repression in human NPCs

Given the variability of Wnt-regulated transcripts across species and cellular contexts, we next defined Wnt-responsive gene sets specific to human iPSC-derived NPCs for use in this and future studies. POU3F2^WT^ and POU3F2^MUT^ NPCs were treated with Wnt3a-conditioned or control media for 24 h, followed by RNA-seq to identify genes significantly upregulated or downregulated with Wnt3a treatment (Fig. 2E and F and Supplementary Tables 5 and 6). Using ranked summary statistics from this analysis (-log_10_ adjusted *P*-value × log_2_ fold change), we defined Wnt-activated (top 100 upregulated) and Wnt-repressed (top 100 downregulated) gene sets for use in subsequent analyses to quantify canonical Wnt signalling as a complementary method to the SUPERTOPFLASH assay (Supplementary Table 7). We then performed GSEA using these Wnt signatures on differential expression from POU3F2^WT^ versus POU3F2^MUT^ NPCs (Supplementary Table 8). POU3F2^MUT^ NPCs showed downregulation of Wnt-activated genes (Fig. 2G and H) and upregulation of Wnt-repressed genes (Fig. 2I and J), validating reduced canonical Wnt signalling via an orthogonal method.

### POU3F2^MUT^ NPCs do not exhibit gross differentiation defects

**Figure 3:**
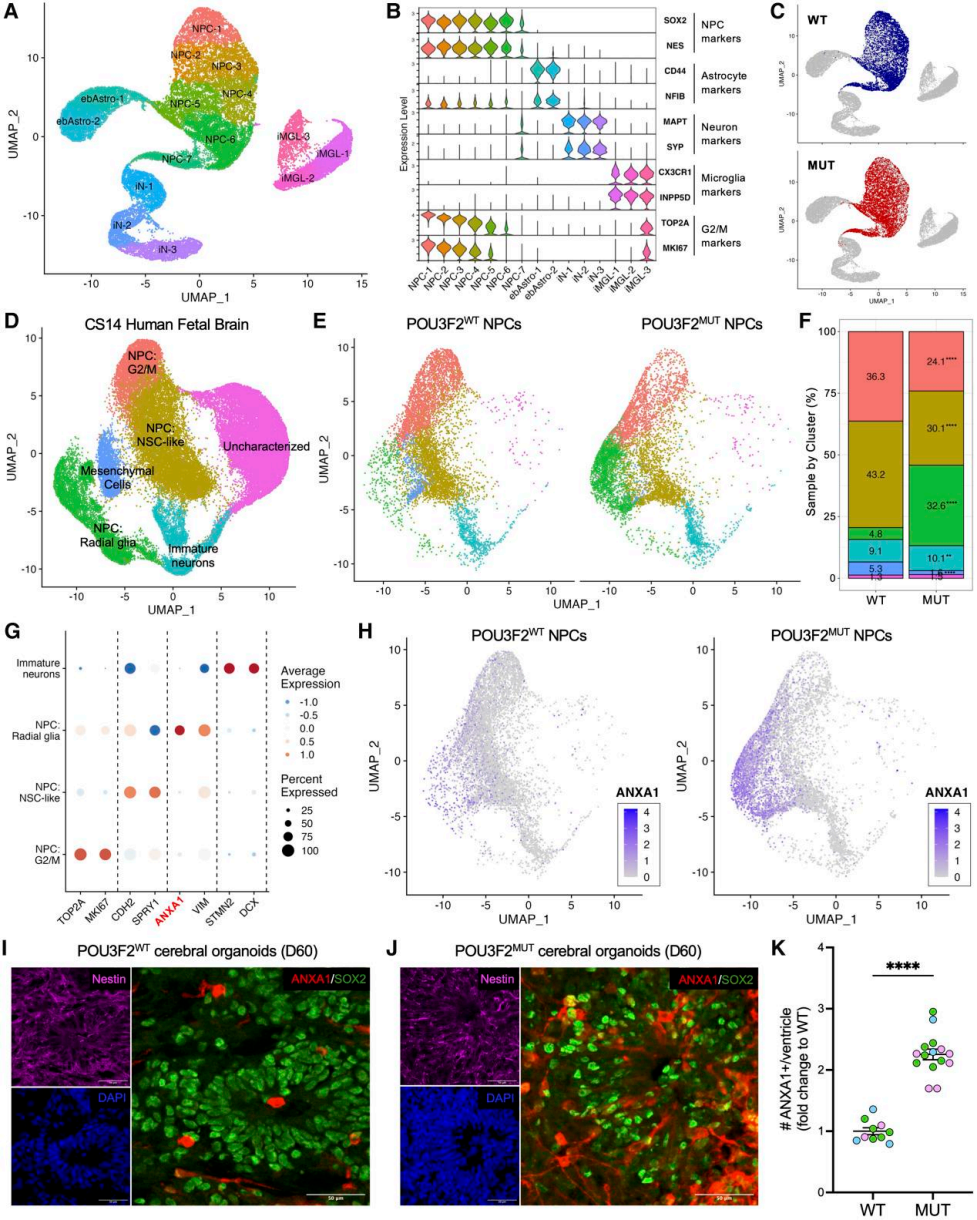
**POU3F2^MUT^ NPCs are shifted toward radial glial identity at the expense of G2/M and neural stem cell-like progenitors.** (**A**) scRNA-seq data of POU3F2^WT^ and POU3F2^MUT^ NPCs were generated and integrated with snRNA-seq of iPSC-derived neurons, astrocytes and microglia. UMAP of integrated data, coloured and labelled by cluster. (**B**) Cell type markers across clusters of iPSC-derived brain cell types. (**C**) UMAP of iPSC-derived brain cell types, with cells from wild-type or mutant NPC cultures highlighted. (**D**) scRNA-seq data of CS14 human fetal brain tissue^73^ (∼5 post-conception weeks) were clustered and labelled according to cell type (see Supplementary Fig. 3B). (**E**) UMAP of POU3F2^WT^ and POU3F2^MUT^ NPCs integrated with CS14 fetal brain data, coloured by cluster. (**F**) Per cent cell composition within each cluster for wild-type and mutant NPCs. Chi-squared test, **P < 0.01, ****P < 0.0001. (**G**) Scaled expression of cluster markers from POU3F2^WT^ and POU3F2^MUT^ NPCs. **(H)** UMAP of POU3F2^WT^ and POU3F2^MUT^ NPCs integrated with CS14 fetal brain data, coloured by *ANXA1* expression. (**I** and **J**) Immunohistochemistry of Nestin (NPC marker), SOX2 (NPC marker) and ANXA1 (radial glia progenitor marker) in (**I**) POU3F2^WT^ and (**J**) PO U3F2^MUT^ Day 60 (D60) cerebral organoids. Scale bar = 50 μm. (**K**) Quantification of the number of ANXA1+ cells per ventricle in POU3F2^WT^ and POU3F2^MUT^ cerebral organoids, mean ± SEM (*n* = 10–15 organoids per genotype). Student's *t*-test, ****P < 0.0001. scRNA-seq = single-cell RNA-sequencing; WT = wild-type; MUT = mutant; NPC = neural progenitor cell; iPSC = induced pluripotent stem cell; UMAP = uniform manifold approximation and projection; SEM = standard error of the mean.

Canonical Wnt signalling is well-characterized as regulating progenitor expansion versus differentiation in early neurodevelopment. To assess how POU3F2 loss affects NPC composition, we performed scRNA-seq of POU3F2^WT^ and POU3F2^MUT^ NPCs. These data were integrated with snRNA-seq of iPSC-derived neurons, astrocytes, and microglia and visualized in UMAP space (Fig. 3A and Supplementary Fig. 2A). Clusters were annotated using cell-type markers: NPCs (SOX2/NES), astrocytes (CD44/NFIB), neurons (MAPT/SYP), microglia (CX3CR1/INPP5D) and G2/M cells (TOP2A/MKI67) (Fig. 3B and Supplementary Fig. 2B). The vast majority of POU3F2^WT^ and POU3F2^MUT^ cells belonged to SOX2+/NES+ clusters (Supplementary Fig. 2C). Immunostaining confirmed no differences in the proportion of PAX6+, Nestin+, MAP2+ or CD44+ cells across *POU3F2* genotypes (Supplementary Fig. 2D and E). These results indicate that POU3F2^MUT^ NPCs do not spontaneously differentiate in the FGF2-supplemented culture conditions utilized here, allowing for the interrogation of POU3F2-relevant mechanisms active in progenitor states without the confounding effects of stochastically generated neuronal/astrocytic populations. Notably, visualization of POU3F2^WT^ versus POU3F2^MUT^ cells suggested a sub-NPC identity shift that prompted further investigation (Fig. 3C).

### POU3F2^MUT^ NPCs demonstrate a shift toward radial glia progenitor fate

POU3F2^WT^ and POU3F2^MUT^ NPC scRNA-seq data were integrated with scRNA-seq data of human fetal brain at Carnegie Stage 14 (CS14, ∼5 weeks post-conception)^73^ (Fig. 3D and E and Supplementary Table 9). Other datasets from this study (CS13-CS19) were analysed; CS14 was chosen due to the large number of high-quality cells, the prevalence of progenitor diversity and the degree of alignment with our NPC dataset. We identified three progenitor clusters marked by *SOX2* and *NES* expression (Supplementary Fig. 2F and G and 3A), with subtype identity assigned based on marker expression in the CS14 human fetal brain (Supplementary Fig. 3B). The progenitor clusters were assigned as: (i) cycling G2/M progenitors; (ii) neuroepithelial/neural stem cell-like (NSC-like) progenitors; and (iii) radial glia progenitors (Supplementary Fig. 3C–J). POU3F2^MUT^ NPC cultures displayed reduced proportions of G2/M and NSC-like progenitors, with a corresponding increase in radial glia progenitors (Fig. 3F–H). Pseudotime analysis further indicated that POU3F2^MUT^ NPCs exhibit a more developmentally advanced state, consistent with premature specification of radial glia (Supplementary Fig. 3K and L). However, radial glial cells are often assigned not only by molecular signatures but also by their morphology and localization in the developing brain. Given that our NPC monolayer cultures cannot adequately report this, we next validated this phenotype using 3D cerebral organoid models. Immunostaining for ANXA1 showed increased radial glia per ventricle in POU3F2^MUT^ organoids across all genetic backgrounds (Fig. 3I–K). Although ANXA1 is exclusively expressed by radial glia in our NPCs and in the fetal brain (Fig. 3H and Supplementary Fig. 3I) and has been previously shown to label ventricular zone-enriched radial glia,^89^ we further confirmed this phenotype using canonical radial glia markers VIM and BLBP (Supplementary Fig. 3M). While VIM is broadly expressed in all NPC subtypes (Supplementary Fig. 3C–E), quantification of high-expressing VIM+ or BLBP+ cells mirrored the ANXA1-based results (Supplementary Fig. 3N and O), supporting our finding that *POU3F2* loss-of-function results in increased radial glia specification.

### Temporal analyses show Wnt signalling disruption precedes shift in radial glial fate in POU3F2^MUT^ NPCs

**Figure 4:**
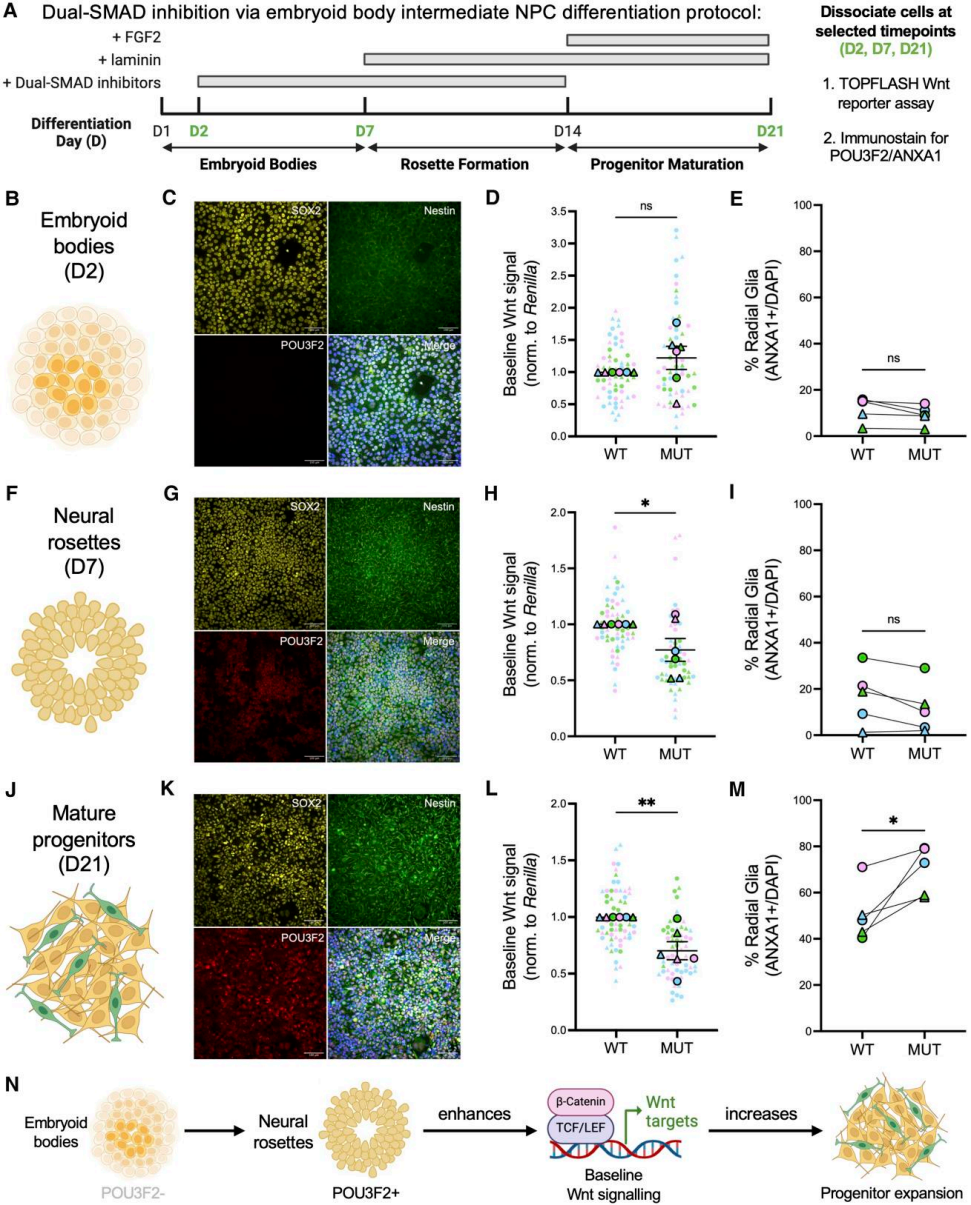
**Time-course experiments suggest that the onset of POU3F2 expression alters baseline Wnt signalling, which then impacts progenitor specification**. (**A**) Schematic of time-course experiment across stages of the dual-SMAD inhibition via embryoid body intermediate NPC differentiation protocol. At each indicated time point, cells were dissociated and subjected to analysis of the onset of POU3F2 expression, baseline canonical Wnt signalling and progenitor specification to radial glia. (**B**, **F** and **J**) Description and representative schematic of each time point. (**C**, **G** and **K**) Immunocytochemistry of SOX2, Nestin and POU3F2 in POU3F2^WT^ NPCs. Scale bar = 100 μm. (**D, H** and **L**) Baseline canonical Wnt signalling, as measured by a SUPERTOPFLASH assay, in POU3F2^WT^ and POU3F2^MUT^ NPCs, mean ± SEM (*n* = 6 differentiations). Paired *t*-test, ns = not significant, *P < 0.05, **P < 0.01. (**E**, **I** and **M**) Quantification of ANXA1+/DAPI (%) in POU3F2^WT^ and POU3F2^MUT^ NPCs, mean ± SEM (*n* = 5 differentiations). Paired *t*-test, ns = not significant, *P < 0.05. (**N**) Model of POU3F2-mediated regulation of baseline canonical Wnt signalling, which then results in alterations of progenitor specification. Schematics created in BioRender. Benoit, C. (2025) https://BioRender.com/5nz5qvx. MUT = mutant; NPC = neural progenitor cell; SEM = standard error of the mean; SMAD = Suppressor of Mothers Against Decapentaplegic; WT = wild-type.

To clarify the temporal relationship between Wnt signalling and altered progenitor fate in POU3F2^MUT^ NPCs, we performed a time-course analysis across key stages of differentiation (Fig. 4A and Supplementary Fig. 4). The selected time points represent stages of NPC differentiation: embryoid bodies (D2; Fig. 4B–E), neural rosettes (D7; Fig. 4F–I) and late-stage progenitors (D21; Fig. 4J–M). At each stage, cells were immunostained for POU3F2 (to assess onset of POU3F2 expression) and ANXA1 (to assess radial glial specification) or subjected to the SUPERTOPFLASH assay to quantify baseline Wnt signalling. POU3F2 expression in POU3F2^WT^ NPCs and reduced Wnt signalling in POU3F2^MUT^ NPCs were first observed at D7, preceding the increase in radial glial specification in POU3F2^MUT^ NPCs detected by D21. These results support the hypothesis that POU3F2 expression increases baseline canonical Wnt signalling, which then affects the expansion versus specification of neural progenitors (Fig. 4N).

### *POU3F2* levels associate with Wnt signalling in a large cohort of genetically diverse NPCs

**Figure 5:**
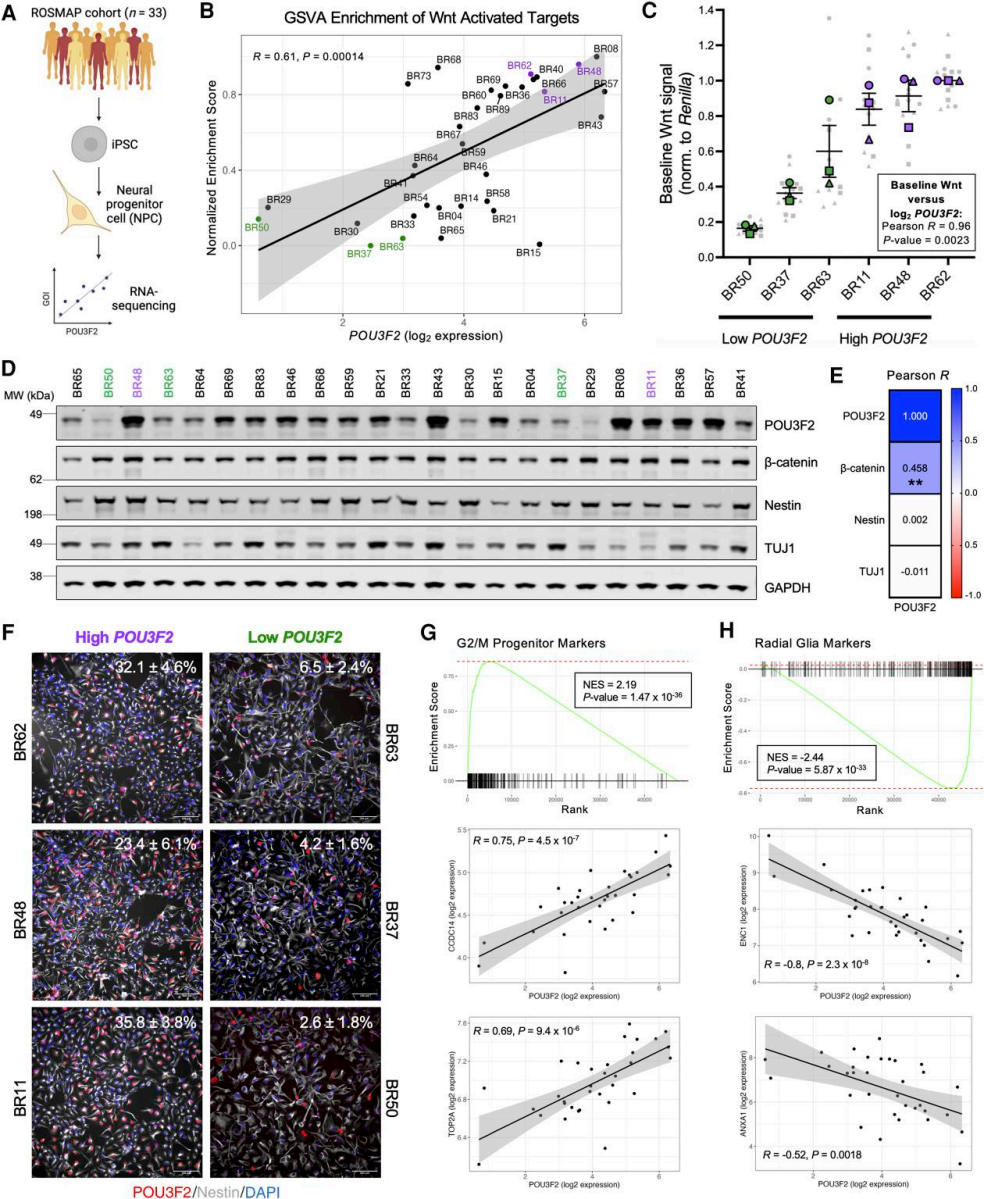
**Natural genetic variation of *POU3F2* levels across a cohort of human NPCs reveals that reduced *POU3F2* levels are associated with reduced baseline canonical Wnt signalling and increased expression of radial glia markers**. (**A**) Schematic of experimental design for RNA-seq of iPSC-derived NPCs from participants in the ROSMAP cohorts. (**B**) GSVA enrichment of Wnt-activated targets (top 100, ranked by -log_10_ adjusted *P*-value * log_2_ fold change) across participant-derived NPCs. Participants with high or low *POU3F2* expression are highlighted. Pearson correlation coefficient and *P*-value shown. (**C**) Baseline canonical Wnt signalling, as measured by a SUPERTOPFLASH assay, in participant-derived NPCs exhibiting high or low *POU3F2* expression, mean ± SEM (*n* = 3 differentiations). (**D**) Western blot analysis of NPCs demonstrating natural variation in POU3F2 levels across divergent genetic backgrounds: POU3F2, β-catenin, Nestin (NPC marker), TUJ1 (neuron marker) and GAPDH (loading control) are shown. Representative image (*n* = 23 lines) shown. (**E**) Pearson correlation of POU3F2 with β-catenin, Nestin and TUJ1. Value inside box corresponds to correlation coefficient, **P < 0.01 (*n* = 2 wells per line, 33 lines). (**F**) Immunocytochemistry of POU3F2, Nestin and DAPI in selected participant-derived NPCs. Per cent of POU3F2+/DAPI cells labelled in top right of each image, mean ± SD (*n* = 20 fields per well, 1 well per line). Scale bar = 100 μm. **(G–H)** GSEA of genes correlated with *POU3F2* using custom gene sets of (**G**) G2/M progenitor markers or (**H**) radial glia markers. Pearson correlation of selected leading-edge G2/M progenitor or radial glia markers are shown. Schematics created in BioRender. Benoit, C. (2025) https://BioRender.com/5nz5qvx. DAPI = 4',6-diamidino-2-phenylindole; GSEA = gene set enrichment analysis; GSVA = gene set variation analysis; iPSC = induced pluripotent stem cell; NPC = neural progenitor cell; ROSMAP = Religious Orders Study/Memory and Aging Project; SD = standard deviation; SEM = standard error of the mean.

Our findings reveal a novel role for POU3F2 in regulating canonical Wnt signalling and progenitor identity in human NPCs. However, biallelic *POU3F2* loss-of-function is not observed in humans, suggesting that disease-relevant effects may arise from more subtle variation in POU3F2 levels. To explore this, we performed bulk RNA-seq of iPSC-derived NPCs from 33 genetically diverse individuals in the Religious Orders Study/Memory and Aging Project (ROSMAP) cohorts^46-49^ which spanned the spectrum of cognitive ability in adulthood (Fig. 5A and Supplementary Tables 10 and 11). Gene set variation analysis (GSVA) showed that enrichment of Wnt-activated targets positively associated with *POU3F2* expression, indicating that natural variation in *POU3F2* levels can modulate canonical Wnt signalling (Fig. 5B). To validate this, we performed the SUPERTOPFLASH assay in NPCs from six individuals (three high- and three low-*POU3F2* expressers), confirming a positive relationship between *POU3F2* expression and baseline Wnt activity (Fig. 5C). Immunostaining showed no association between *POU3F2* levels and markers of broad cell fate (PAX6, Nestin, MAP2, CD44) (Supplementary Fig. 5A). Protein lysates across all lines revealed a significant correlation between POU3F2 and β-catenin abundance, but not with Nestin or TUJ1 (Fig. 5D and E). These results support a model in which POU3F2 activates canonical Wnt signalling in NPCs but does not alter broad cell fate. Immunocytochemistry of POU3F2 showed variation in the per cent of cells with high nuclear POU3F2 expression, suggesting heterogeneous POU3F2 expression among NPC subtypes (Fig. 5F). We next compared our POU3F2^WT^ lines (YZ1/BR33/BR24) to the ROSMAP cohort and found that the three lines chosen for mutant studies are well-distributed on the spectrum of POU3F2 expression (Supplementary Fig. 5B). Finally, we accessed fetal forebrain data from the Human Developmental Biology Resource (HDBR) to compare POU3F2 expression between individuals across developmental time. These data reveal that POU3F2 expression is highly dynamic and variable across individuals at the same developmental stage, echoing the heterogeneity seen in our model (Supplementary Fig. 5C).

### Lower *POU3F2* levels associate with elevated radial glia marker expression

To determine whether natural variation in *POU3F2* expression influences progenitor identity in genetically diverse NPCs, we performed genome-wide expression association with *POU3F2* and generated a ranked gene list (-log_10_  *P*-value × Pearson coefficient) (Supplementary Table 12). GSEA was performed, queried against G2/M and radial glia markers defined in NPCs that clustered with these subtypes in human fetal brain (Supplementary Table 9). Genes positively correlated with *POU3F2* expression were enriched for G2/M markers (Fig. 5G), while genes negatively correlated with *POU3F2* expression were enriched for radial glia markers (Fig. 5H). Immunostaining of high- and low-*POU3F2* expressing lines further confirmed this association, with per cent positivity of ANXA1 and BLBP negatively correlating with *POU3F2* expression (Supplementary Fig. 5D). These findings align with our POU3F2^MUT^ phenotypes and support a model in which lower *POU3F2* expression induces a shift toward radial glial identity.

### Identification of POU3F2-binding partners and direct transcriptional targets

**Figure 6:**
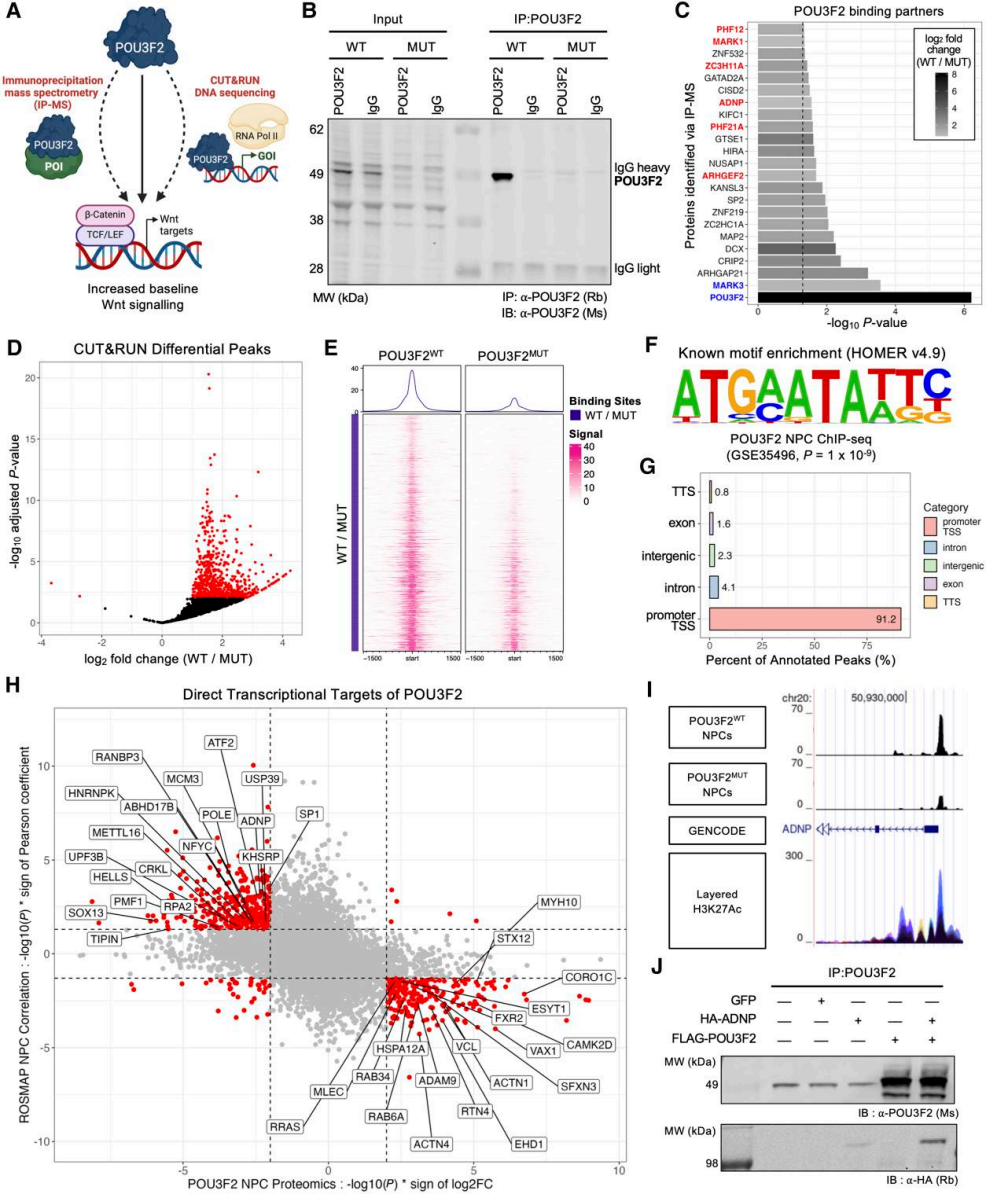
**Identification of downstream effector proteins of POU3F2 function through the characterization of binding partners and transcriptional targets of POU3F2.** (**A**) Schematic of methodology to identify potential effectors of POU3F2 capable of modulation of the Wnt signalling pathway. (**B**) Immunoprecipitation of POU3F2 or IgG (negative control) in POU3F2^WT^ and POU3F2^MUT^ NPCs, followed by western blot analysis. (**C**) Differentially abundant proteins between IP-MS samples of POU3F2^WT^ and POU3F2^MUT^ NPCs (*n* = 3 samples per line). Highlighted genes have been implicated in autism (red = SFARI gene, blue = GWAS risk locus). (**D**) CUT&RUN was performed to identify direct transcriptional targets of POU3F2 in human NPCs. Volcano plot of differentially bound peaks (WT / MUT). Significance threshold = peaks with adjusted *P*-value < 0.01 (Input = 2 sample per line, CUT&RUN = 2 samples per line). (**E**) Profile plot of peak enrichment in POU3F2^WT^ versus POU3F2^MUT^ cells. (**F**) Known motif analysis showing enrichment of a motif identified in an independent POU3F2 ChIP-seq dataset (GSE35496).^90^(**G**) Per cent of differentially bound peaks by annotation (TTS = transcription termination site, TSS = transcription start site).(**H**) Identification of candidate POU3F2 effector genes, based upon the following criteria: (i) evidence of direct regulation by POU3F2 through CUT&RUN; (ii) positive correlation with *POU3F2* expression in participant-derived NPCs; and (iii) protein downregulated in POU3F2^MUT^ NPCs. Genes that meet these criteria are labelled on the integration plot. (**I**) UCSC Genome Browser showing the following tracks: bedGraph CUT&RUN read pileups, GENCODE v44 gene annotation, and H3K27Ac mark from ENCODE. (**J**) Immunoprecipitation of POU3F2 in HEK293T cells overexpressing HA-ADNP (lane 3), FLAG-POU3F2 (lane 4) or both (lane 5), followed by western blot analysis. IB = immunoblot. Schematics created in BioRender. Benoit, C. (2025) https://BioRender.com/35q22ly. ChIP = chromatin immunoprecipitation; CUT&RUN = cleavage under targets and release using nuclease; ENCODE = encyclopedia of DNA elements; GWAS = genome-wide association study; HA = haemagglutinin; IP-MS = immunoprecipitation mass spectrometry; MUT = mutant; NPC = neural progenitor cell; SEM = standard error of the mean; SFARI = Simons Foundation Autism Research Initiative; WT = wild-type.

To identify effectors of POU3F2 capable of Wnt pathway modulation, we performed: (i) co-immunoprecipitation followed by TMT-MS to define POU3F2-interacting partners in human NPCs; and (ii) CUT&RUN to define direct transcriptional targets of POU3F2 in human NPCs (Fig. 6A). For both analyses, POU3F2^MUT^ NPCs were analysed in parallel as negative controls. Western blot confirmed efficient POU3F2 pull-down in wild-type but not IgG or mutant samples (Fig. 6B). TMT-MS identified 23 proteins significantly enriched in POU3F2^WT^ NPCs (Fig. 6C; Supplementary Table 13). Interacting proteins were enriched for those implicated in ASD through genetic studies (chi-square test, *P* < 0.0001), including the high-confidence ASD risk gene, *ADNP*.^91^ Four interacting proteins (DCX, PHF21A, ADNP, PHF12) have been associated with other neurodevelopmental disorders, as defined by the Developmental Brain Disorders Database.^92^ Further, ADNP^93,94^ and MARK1^95,96^ have been previously implicated in canonical Wnt signalling. In parallel to this method, CUT&RUN was performed to identify regions of the genome where POU3F2 binds in human NPCs. This analysis revealed differentially bound peaks in POU3F2^WT^ NPCs (Fig. 6D and E; Supplementary Table 14), which were enriched for a known POU3F2 DNA binding motif^90^ (Fig. 6F) and predominantly localized to promoter regions (Fig. 6G). Notably, genes upregulated with Wnt activation were significantly enriched in POU3F2 target genes (chi-square test, *P* < 0.0001), while genes downregulated with Wnt activation were not (chi-square test, *P* = 0.94). We next prioritized POU3F2 effector candidates meeting these criteria: (i) evidence of POU3F2-mediated transcription; (ii) positive correlation with *POU3F2* expression; and (iii) downregulation in POU3F2^MUT^ NPCs. Nineteen candidate positive effectors of POU3F2 were identified using this strategy (Fig. 6H; upper left quadrant), including ADNP, which was identified as both a direct transcriptional target (Fig. 6I) and binding partner (Fig. 6C) of POU3F2 in human NPCs. Given that ADNP is a high-confidence ASD gene mutated in syndromic autism,^91^ we validated the ADNP–POU3F2 interaction via co-immunoprecipitation in HEK293T cells (Fig. 6J).

### POU3F2 transcriptionally activates *SOX13* and *ADNP* to regulate canonical Wnt signalling

**Figure 7:**
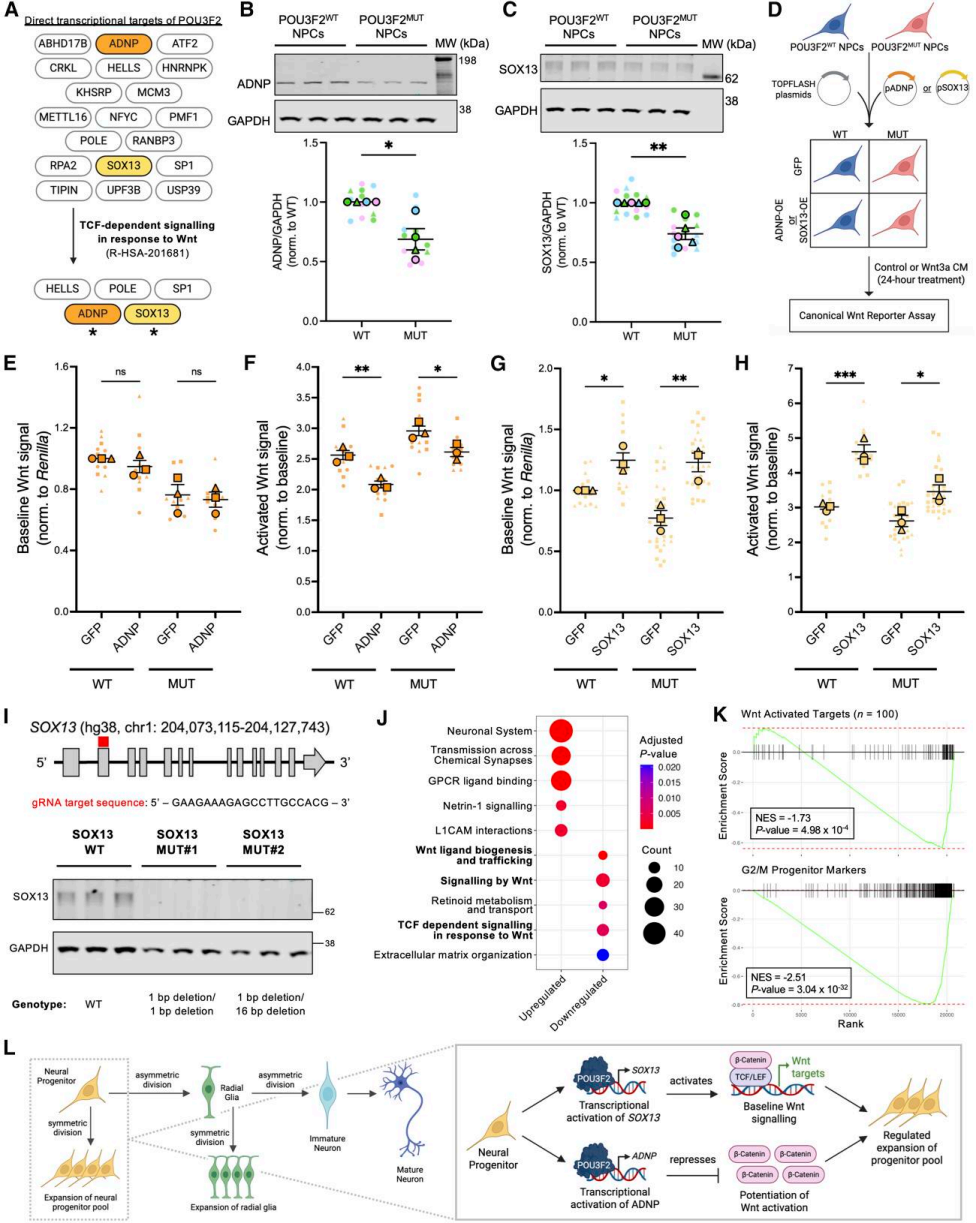
**POU3F2 activates expression of *SOX13* and *ADNP* to regulate canonical Wnt signalling in NPCs.** (**A**) Potential direct effectors of POU3F2 were prioritized if they belonged to the TCF-dependent signalling in response to Wnt Reactome pathway (R-HSA-201681). (**B**) Quantification of ADNP protein expression measured via western blotting in POU3F2^WT^ and POU3F2^MUT^ NPCs, mean ± SEM (*n* = 4 differentiations). Paired *t*-test, *P < 0.05. (**C**) Quantification of SOX13 protein expression measured via western blotting in POU3F2^WT^ and POU3F2^MUT^ NPCs, mean ± SEM (*n* = 5 differentiations). Paired *t*-test, **P < 0.01. (**D**) Schematic of experimental design for overexpression of a gene of interest followed by the SUPERTOPFLASH canonical Wnt reporter assay. (**E**) Baseline canonical Wnt signalling, as measured by a SUPERTOPFLASH assay, in POU3F2^WT^ and POU3F2^MUT^ NPCs expressing GFP (negative control) or ADNP, mean ± SEM (*n* = 3 differentiations). Mixed-effects model with *post hoc* Sidak's test, ns = not significant. (**F**) Activated canonical Wnt signalling, as measured by a SUPERTOPFLASH assay, in POU3F2^WT^ and POU3F2^MUT^ NPCs expressing GFP (negative control) or ADNP, mean ± SEM (*n* = 3 differentiations). Mixed-effects model with *post hoc* Sidak's test, *P < 0.05, **P < 0.01. (**G**) Baseline canonical Wnt signalling, as measured by a SUPERTOPFLASH assay, in POU3F2^WT^ and POU3F2^MUT^ NPCs expressing GFP (negative control) or SOX13, mean ± SEM (*n* = 3 differentiations). GFP control data represented here is included in Fig. 2B. Mixed-effects model with *post hoc* Sidak's test, *P < 0.05, **P < 0.01. (**H**) Activated canonical Wnt signalling, as measured by a SUPERTOPFLASH assay, in POU3F2^WT^ and POU3F2^MUT^ NPCs expressing GFP (negative control) or SOX13, mean ± SEM (*n* = 3 differentiations). GFP control data represented here is included in Fig. 2C. Mixed-effects model with *post hoc* Sidak's test, *P < 0.05, ***P < 0.001. (**I**) Genomic structure of *SOX13*. Box above gene indicates relative position of gRNA target sequence. Western blot validation of SOX13 knockdown (GAPDH = loading control). (**J**) Gene ontology pathway enrichment results of differential gene expression in SOX13^MUT^ NPCs, separated by directionality of log_2_ fold change. (**K**) GSEA of Wnt-activated targets and G2/M progenitor markers in SOX13^MUT^ versus SOX13^WT^ differential expression. (**L**) Model of POU3F2-mediated regulation of canonical Wnt signalling and progenitor expansion via transcription of *ADNP* and *SOX13*. Schematics created in BioRender. Benoit, C. (2025) https://BioRender.com/35q22ly, https://BioRender.com/baxexcu. gRNA = guide RNA; GSEA = gene set enrichment analysis; MUT = mutant; NPC = neural progenitor cell; SEM = standard error of the mean; WT = wild-type.

From the list of 19 candidate POU3F2 effectors, we prioritized five that have been previously associated with Wnt signalling (R-HSA-201681^85^): *HELLS*, *POLE*, *SP1*, *ADNP* and *SOX13* (Fig. 7A). Of these, we focused on *ADNP* (for reasons above) and *SOX13,* given evidence of SOX/POU transcription factor co-regulation^90,97,98^ and prior evidence of SOX13-mediated regulation of Wnt signalling.^99-101^ We first confirmed expression of SOX13, ADNP and β-catenin in POU3F2^WT^ NPCs via immunostaining (Supplementary Fig. 6A–C). We then accessed the HDBR and BrainSpan^102^ databases to assess expression of *POU3F2, SOX13, ADNP* and *CTNNB1* (encoding β-catenin) across developmental time in human fetal cortical tissue (Supplementary Fig. 6D and E). Additionally, we examined their expression in CS14 human fetal brain (Supplementary Fig. 6F). These analyses show that *POU3F2*, *SOX13*, *ADNP* and *CTNNB1* are expressed in NPCs and human brain at developmental time points relevant to this study. We next validated that ADNP and SOX13 protein levels are reduced in POU3F2^MUT^ NPCs (Fig. 7B and C). To assess functional roles, we overexpressed ADNP, SOX13 or GFP (negative control) in POU3F2^WT^ and POU3F2^MUT^ NPCs, followed by the SUPERTOPFLASH assay (Fig. 7D). ADNP overexpression did not affect baseline Wnt signalling (Fig. 7E) but significantly reduced Wnt3a responsiveness in both genotypes (Fig. 7F). In contrast, SOX13 overexpression enhanced baseline Wnt signalling in POU3F2^WT^ NPCs and rescued Wnt signalling defects in POU3F2^MUT^ NPCs (Fig. 7G). SOX13 overexpression also increased Wnt3a responsiveness in both genotypes (Fig. 7H). Additionally, SOX13 overexpression in POU3F2^MUT^ NPCs reduced ANXA1 expression without altering POU3F2 levels, consistent with SOX13 acting downstream of POU3F2 (Supplementary Fig. 7A–D). Together, these findings support SOX13 as the primary effector of POU3F2 driving canonical Wnt signalling, while ADNP may temper Wnt potentiation to allow for fine-tuned control of progenitor expansion.

### Knockdown of *SOX13* phenocopies loss of POU3F2 in human NPCs

To determine whether SOX13 mediates the Wnt signalling deficits seen in POU3F2^MUT^ NPCs, we performed CRISPR/Cas9 mutagenesis to generate *SOX13* loss-of-function lines. One unedited SOX13^WT^ and two SOX13^MUT^ clones with compound heterozygous, protein-disrupting mutations were selected for downstream analysis. iPSCs were differentiated into NPCs and assessed for markers of NPC, neuronal and astrocytic fate (Supplementary Fig. 7E). Western blot analysis confirmed loss of SOX13 protein in SOX13^MUT^ NPCs (Fig. 7I). Bulk RNA-seq revealed transcriptomic changes in SOX13^MUT^ NPCs that mirrored those in POU3F2^MUT^ NPCs, including downregulation of pathways related to canonical Wnt signalling (Fig. 7J and Supplementary Tables 15 and 16). GSEA further showed significant downregulation of Wnt-activated targets and G2/M progenitor markers (Fig. 7K), consistent with a phenocopy of *POU3F2* loss-of-function. These results, together with those from overexpression studies, support a model in which SOX13 and ADNP act downstream of POU3F2 to modulate Wnt signalling, which in turn enables regulated expansion of the progenitor pool (Fig. 7L).

### Autism-related genes are enriched in datasets relating to POU3F2 function

**Figure 8:**
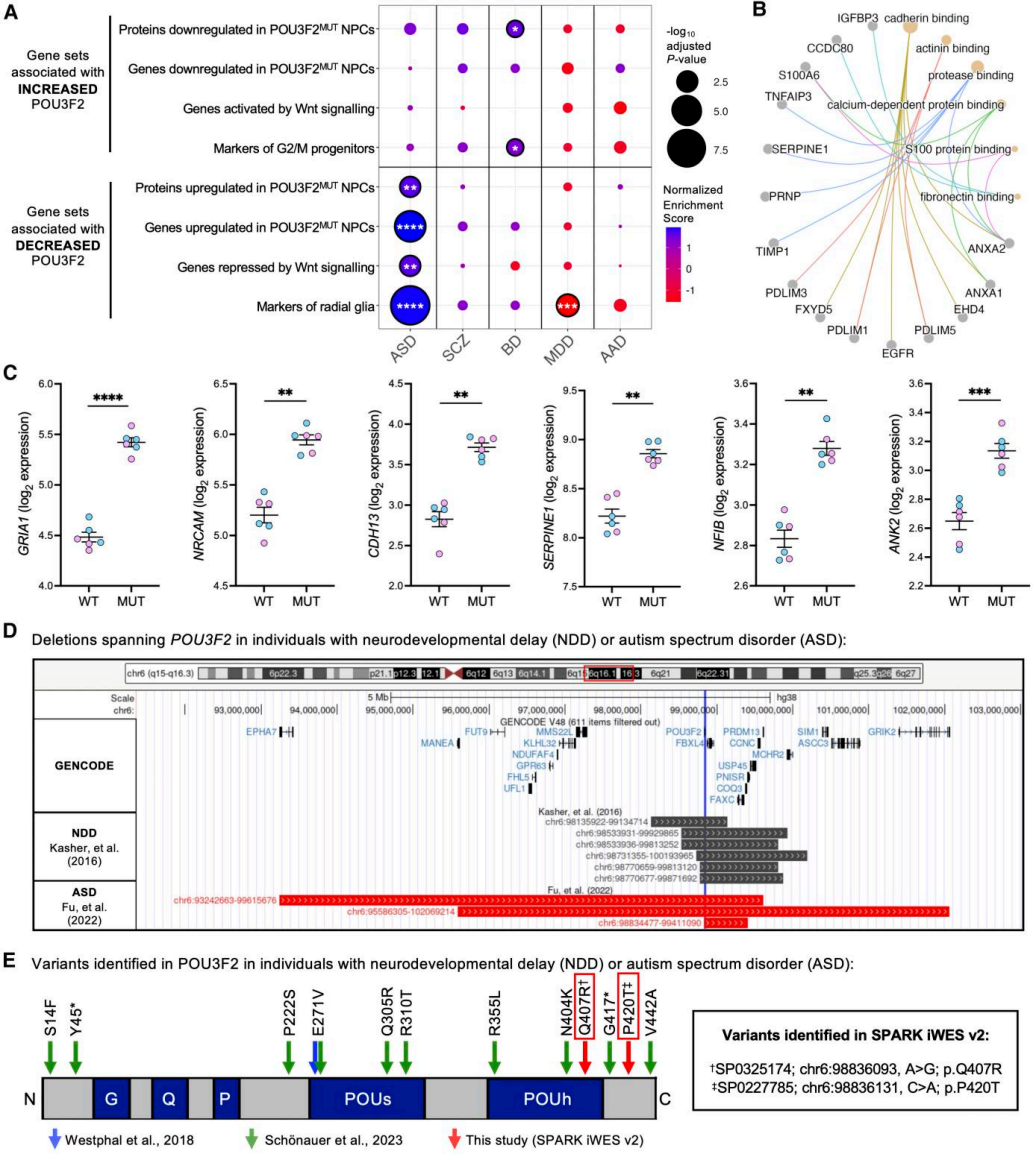
***POU3F2* loss-of-function is implicated in autism spectrum disorder through disrupted ASD gene networks in POU3F2^MUT^ NPCs and through rare-variant identification in individuals with ASD**. (**A**) Gene set enrichment analysis of POU3F2-related gene sets in microarray data from neurodevelopmental and neuropsychiatric disorders.^103^ Outline around circle indicates adjusted *P*-value <0.05. *P < 0.05, **P < 0.01, ***P < 0.001, ****P < 0.0001. (**B**) Network pathway enrichment plot of genes and/or proteins upregulated in POU3F2^MUT^ NPCs and upregulated in autism microarray data. (**C**) Log_2_ batch corrected TPM of selected SFARI genes that are dysregulated in POU3F2^MUT^ NPCs, mean ± SEM. Mixed-effects model, **P < 0.01, ***P < 0.001, ****P < 0.0001. (**D**) Summarization of deletions spanning *POU3F2* that have been associated with neurodevelopmental delay^20^ (NDD) or autism spectrum disorder^104^ (ASD). (**E**) Missense and protein-truncating variants in POU3F2 identified in subjects with NDD or ASD. Details for the identified variants from the SPARK iWES v.2 release are described. iWES = integrated whole exome sequencing; MUT = mutant; NPC = neural progenitor cell; SEM = standard error of the mean; SFARI = Simons Foundation Autism Research Initiative; SPARK = Simons Foundation Powering Autism Research for Knowledge; TPM = transcripts per million; WT = wild-type.

Although this study reveals critical roles for POU3F2 in regulating Wnt signalling and radial glial specification in human NPCs, it remains unclear whether these mechanisms directly contribute to neurodevelopmental deficits observed in individuals with *POU3F2* loss-of-function. To explore potential relevance to human disease, we compared gene networks disrupted in our model to those altered in cortical transcriptomic data from individuals with five major neuropsychiatric disorders.^103^ Using this approach, we found that genes upregulated in ASD brains were strongly and significantly enriched for gene sets associated with decreased POU3F2 activity generated in this study (Fig. 8A). Genes and/or proteins upregulated in POU3F2^MUT^ NPCs and upregulated in ASD were enriched for pathways relating to cell–cell interactions and extracellular matrix composition, processes which warrant future investigation (Fig. 8B). Additionally, high-confidence ASD risk genes were significantly enriched in genes differentially expressed in POU3F2^MUT^ NPCs (Fig. 8C; chi-square test, *P* = 0.027). These findings suggest that POU3F2 dysregulation in NPCs may contribute to ASD-relevant molecular pathways, highlighting the utility of this model in studying neurodevelopmental disease mechanisms.

### Identification of loss-of-function *POU3F2* mutations in individuals with ASD

Based on these findings, we interrogated existing exome sequencing data from subjects with ASD. In the most recent Autism Sequencing Consortium study,^104^ we identified three *de novo* copy number variants disrupting *POU3F2* in individuals with ASD (Fig. 8D; Supplementary Table 17), further supporting prior evidence that heterozygous deletions encompassing *POU3F2* are associated with neurodevelopmental disorders.^20^ In the latest release of whole-exome sequencing from the Simons Powering Autism Research (SPARK) consortium,^105^ we identified two *de novo* missense variants in *POU3F2* in individuals with ASD (Fig. 8E). One variant (p.Q407R) resides within the POU homeodomain and is predicted to be pathogenic, whereas the other (p.P420T) has mixed pathogenicity predictions and is considered likely benign.^106^ These findings expand the catalogue of ASD-associated *POU3F2* variants and reinforce the role of *POU3F2* in conferring risk for neurodevelopmental delay and/or ASD.^22,23^

## Discussion

In this study, we investigated the molecular role of POU3F2 in human neurodevelopment using *POU3F2* loss-of-function in iPSC-derived NPCs. POU3F2^MUT^ NPCs exhibited reduced baseline canonical Wnt signalling and decreased proliferation, resulting in premature specification to radial glia at the expense of G2/M and NSC-like progenitors. Further, we found that natural variation in *POU3F2* levels across genetically diverse individuals positively associates with baseline canonical Wnt signalling and negatively associates with radial glia specification. Using unbiased and functional approaches, we show that POU3F2 regulates expression of *SOX13* and *ADNP*, and that together these proteins mediate POU3F2's effects on canonical Wnt signalling. Collectively, these findings support a model in which POU3F2 activates canonical Wnt signalling via *SOX13* and *ADNP* to regulate NPC expansion during early human neurodevelopment.

Previous work from our group showed that activation of canonical Wnt signalling reduced *POU3F2* expression in human iPSC-derived organoids and NPCs.^15,16^ In contrast, a study in mice found that *Pou3f2* was a direct target of canonical Wnt signalling, and that its expression was enhanced upon Wnt activation.^107^ In conjunction with our findings, these results suggest a bidirectional relationship between POU3F2 and Wnt signalling in a species-dependent manner. Multiple mouse studies have provided insight into Pou3f2's role in neurodevelopment.^33-35,37,39,41,42,107^ However, differences in gene compensation, cell-type diversity, and cortical architecture between rodents and humans limit the translatability of these findings.^108^ Given this, human cellular models of brain development provide a unique opportunity to study molecular mechanisms of disease in a human-specific context, particularly with the use of iPSC technology which allows for the study of a variety of cell types from genetically diverse donors.^109^

GWAS, transcriptome-wide association studies (TWAS) and rare-variant studies have implicated *POU3F2* in neurodevelopmental disorders.^20,22-29^ Its role in schizophrenia and bipolar disorder has been supported based on GWAS and transcriptomic studies.^30,31^ Rare heterozygous mutations in *POU3F2* (including deletions, protein-truncating variants and missense variants) have also been linked to developmental delay and ASD,^20,22,23^ consistent with a high predicted loss-of-function intolerance for *POU3F2* in gnomAD.^110^ Of the 22 characterized individuals with *POU3F2* mutations, 21 presented with neurodevelopmental delay, and in one study,^23^ 7 of 12 were diagnosed with ASD. Notably, obesity and hyperphagia—likely due to hypothalamic dysfunction^20,23^—are disproportionately observed in individuals carrying *POU3F2* mutations. In this study, we report five additional ASD cases with *POU3F2* variants: three with large deletions^104^ and two with missense mutations.^105^ Together with our findings, these studies underscore the critical role of POU3F2 in neurodevelopment and suggest a mechanism linking its disruption to ASD and developmental delay. It is also relevant to consider how natural variation in POU3F2 levels may influence neurodevelopment, even in the absence of neurological diagnoses. In this study, we show that natural, genetically encoded variation in *POU3F2* levels can affect Wnt signalling and progenitor specification. Further studies are needed to define the consequences of altered radial glial specification and to identify genetic variants which drive inter-individual differences in *POU3F2* expression using an even larger cohort of individuals.

Previous studies have shown that SOX13 modulates Wnt signalling in non-neuronal tissues, either as an inhibitor^99,100^ or activator^101^ in a context-dependent manner. *Sox13* knockout mice exhibit severe growth defects, supporting a potential role for Sox13 in promoting proliferation.^100^ In this study, we find that SOX13 serves as a major downstream effector of POU3F2 to enhance both baseline and activated canonical Wnt signalling in human NPCs. In addition to SOX13, we also identify ADNP as a second POU3F2 effector. ADNP has diverse roles in embryogenesis,^111^ chromatin reorganization and transcriptional regulation,^112,113^ and neuronal maturation.^114^ Mutations in *ADNP* cause Helsmoortel-Van der Aa syndrome,^91^ the most common monogenic form of autism (estimated at 0.17% of ASD cases).^115^ Previous work has linked ADNP to Wnt signalling, with contrasting effects across cellular contexts. In mouse neurospheres, *Adnp* loss-of-function decreased β-catenin stabilization and reduced Wnt signalling,^94^ whereas in human colon cancer cells, ADNP overexpression led to these same effects.^93^ Here, we show that ADNP overexpression in human NPCs reduces activation of canonical Wnt signalling, suggesting it functions as an inhibitor of Wnt potentiation in this cellular context. We propose that POU3F2 activates *ADNP* transcription as a negative feedback mechanism to attenuate canonical Wnt activation, mirroring feedback regulators such as *AXIN2* and *DKK1*.^116,117^ We further identify ADNP as a POU3F2-interacting protein in NPCs, suggesting that they may form a complex to co-regulate gene transcription. Given their shared links to syndromic autism, future studies will be critical to explore the mechanistic and therapeutic implications of this interaction.

### Limitations of study

In this study, we used biallelic mutations effectively eliminating POU3F2 protein to understand POU3F2's normal function in neurodevelopment. As a result, the phenotypes we observed may differ from—or be more severe than—those seen in individuals with heterozygous *POU3F2* mutations. Future studies using NPCs derived from individuals with specific heterozygous *POU3F2* mutations are needed to bridge the gap between model systems and patient biology. Nonetheless, data from genetically diverse NPCs presented herein support the hypothesis that *POU3F2* acts in a dose-dependent manner to fine-tune Wnt signalling and progenitor specification. Further, our study provides a snapshot of POU3F2's role in neurodevelopment. Wnt signalling is both tightly regulated and highly dynamic across neurodifferentiation, and POU3F2 may have distinct roles across developmental stages. For instance, a previous study of iPSC-derived NPCs from individuals with idiopathic ASD and macrocephaly found reduced Wnt signalling, lower POU3F2 expression and increased proliferation.^118^ That study used NPCs expanded up to seven passages, which is known to affect neurogenic potential and favour glial differentiation.^50,119,120^ Our analyses were restricted to NPCs at or before the third passage, suggesting that differences in progenitor state and neurogenic potential may explain the discrepancies between findings.

## Supplementary Material

awaf221_Supplementary_Data

## Data Availability

All transcriptomic and genomic datasets generated in this study are deposited in the National Center for Biotechnology Information-Gene Expression Omnibus (NCBI-GEO) with the following identification numbers: GSE294199, GSE294370, GSE294372, GSE294377, GSE294379 and GSE294380. The proteomic data generated in this study is included as a Supplementary table. An interactive data viewer for the datasets presented in this study can be found in a Shiny app: https://youngpearselab.shinyapps.io/pou3f2_npc/.
