# Supplementary material for: POU3F2 regulates canonical Wnt signalling via SOX13 and ADNP to expand the neural progenitor population
Source: Brain. 2025 Jun 11;148(12):4325–44. doi: 10.1093/brain/awaf221 (PMC12677912; doi:10.1093/brain/awaf221)
Supplement: awaf221_Supplementary_Data [file awaf221_supplementary_data.zip › brain-2024-02937-File010.pdf]

Supplementary Figure 1

A

Line ID	Clone	Genotype	Protein Coordinate	Consequence
**BR33** 89yo male	WT	wildtype	NA	NA
	MUT#1	3 bp deletion / 14 bp deletion	p.Thr287del / p.Thr287*	1 aa deletion (in POUs) / Frameshift
	MUT#2	3 bp deletion / 14 bp deletion	p.Thr287del / p.Thr287*	1 aa deletion (in POUs) / Frameshift
**BR24** 86yo female	WT	wildtype	NA	NA
	MUT#1	Homozygous 41 bp deletion + 7 bp insertion	p.Phe286* / p.Phe286*	Frameshift
	MUT#2	Compound heterozygous 1 bp deletions	p.Gln288* / p.Gln288*	Frameshift
**YZ1** 16wo female (IMR90)	WT	wildtype	NA	NA
	MUT#1	1 bp deletion / 24 bp deletion	p.Leu284-Gly292del / p.Lys283*	Frameshift / 8 aa deletion (in POUs)
	MUT#2	1 bp deletion / 1 bp deletion + 3 bp insertion	p.Thr287* / p.Gln288*	Frameshift

B

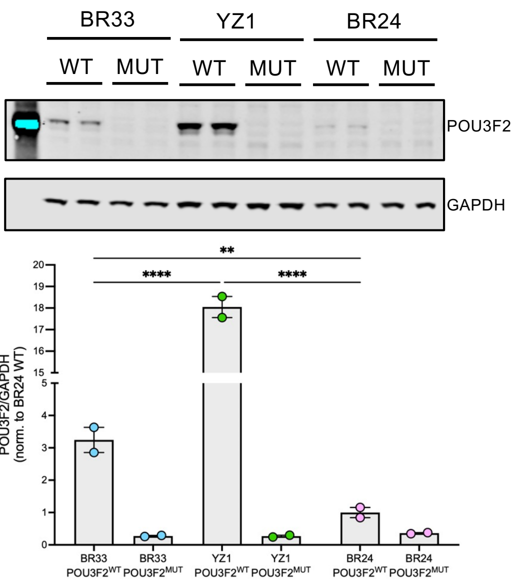

C

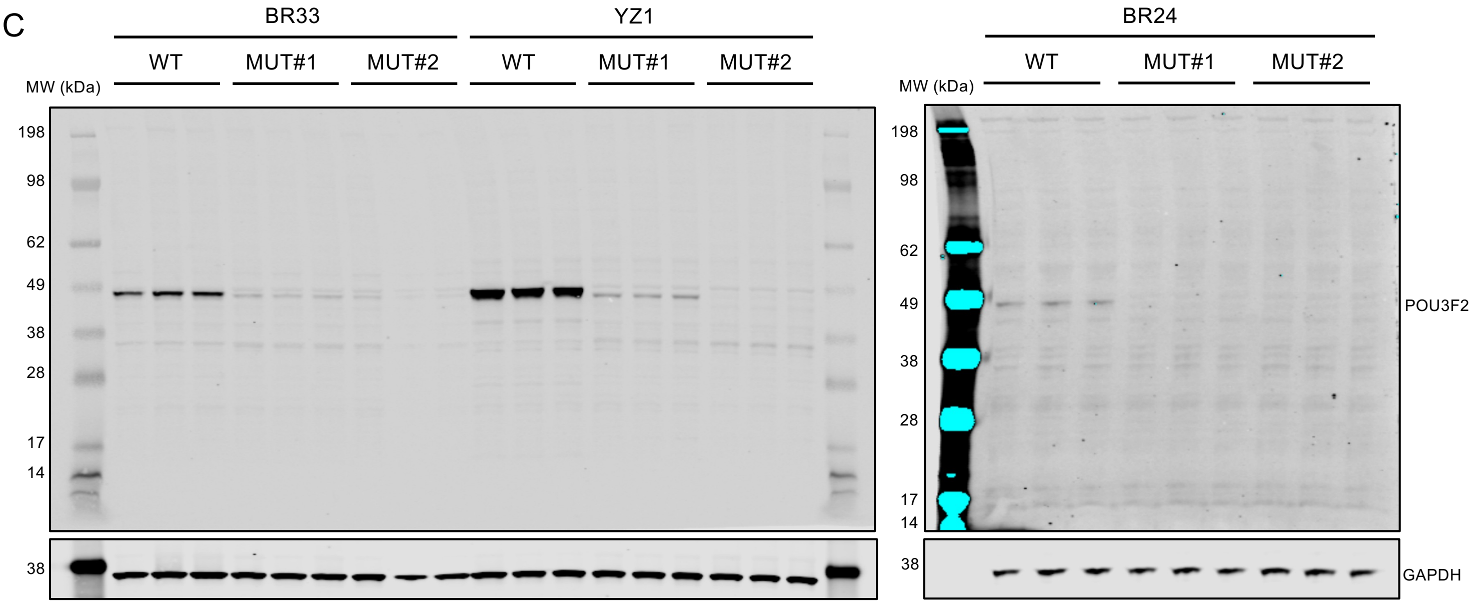

D

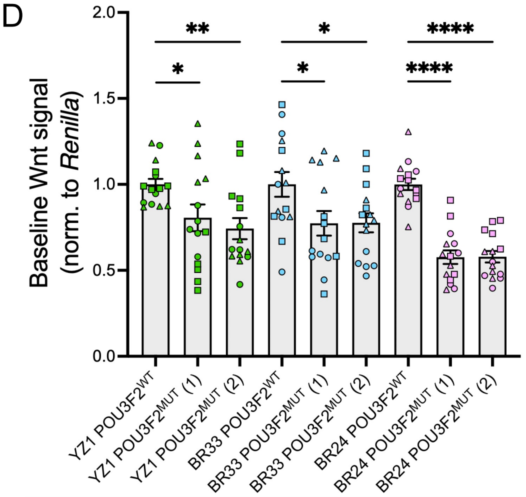

E

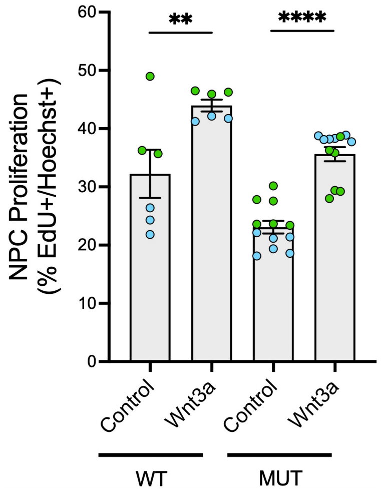

F

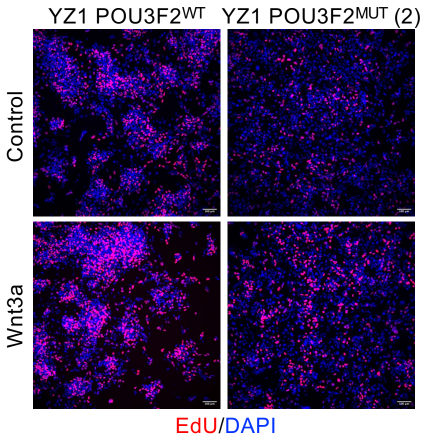

Supplementary Figure 1. Generation and characterization of Wnt signalling and proliferation phenotypes in POU3F2^{MUT} NPCs. (A) Table depicting *POU3F2* genotypes and consequences on protein sequence for each of the mutants used in this study. (B) Quantification of POU3F2 expression across genetic backgrounds of POU3F2^{WT} and POU3F2^{MUT} NPCs, mean ± SEM (n = 2 wells). ANOVA with post-hoc Sidak's test, ***P*<0.01, *****P*<0.0001. (C) Western Blot analysis of POU3F2^{WT} and POU3F2^{MUT} NPCs, probing for POU3F2 and GAPDH. (D) Baseline canonical Wnt signalling, as measured by a SUPERTOPFLASH assay, in POU3F2^{WT} and POU3F2^{MUT} NPCs, split by line (n = 5 wells per differentiation, 3 differentiations per line indicated by shape). ANOVA with post-hoc Dunnett's test, **P*<0.05, ***P*<0.01, *****P*<0.0001. (E) EdU incorporation assay in POU3F2^{WT} and POU3F2^{MUT} NPCs treated with control or Wnt3a conditioned media (n = 9 fields per well, 6-12 wells per category). Control data represented here is included in Figure 2D. ANOVA with post-hoc Sidak's test, ***P*<0.01, *****P*<0.0001. (F) Representative images of EdU incorporation assay, in POU3F2^{WT} and POU3F2^{MUT} NPCs treated with control or Wnt3a conditioned media. Scale bar = 100 μm.

Supplementary Figure 2

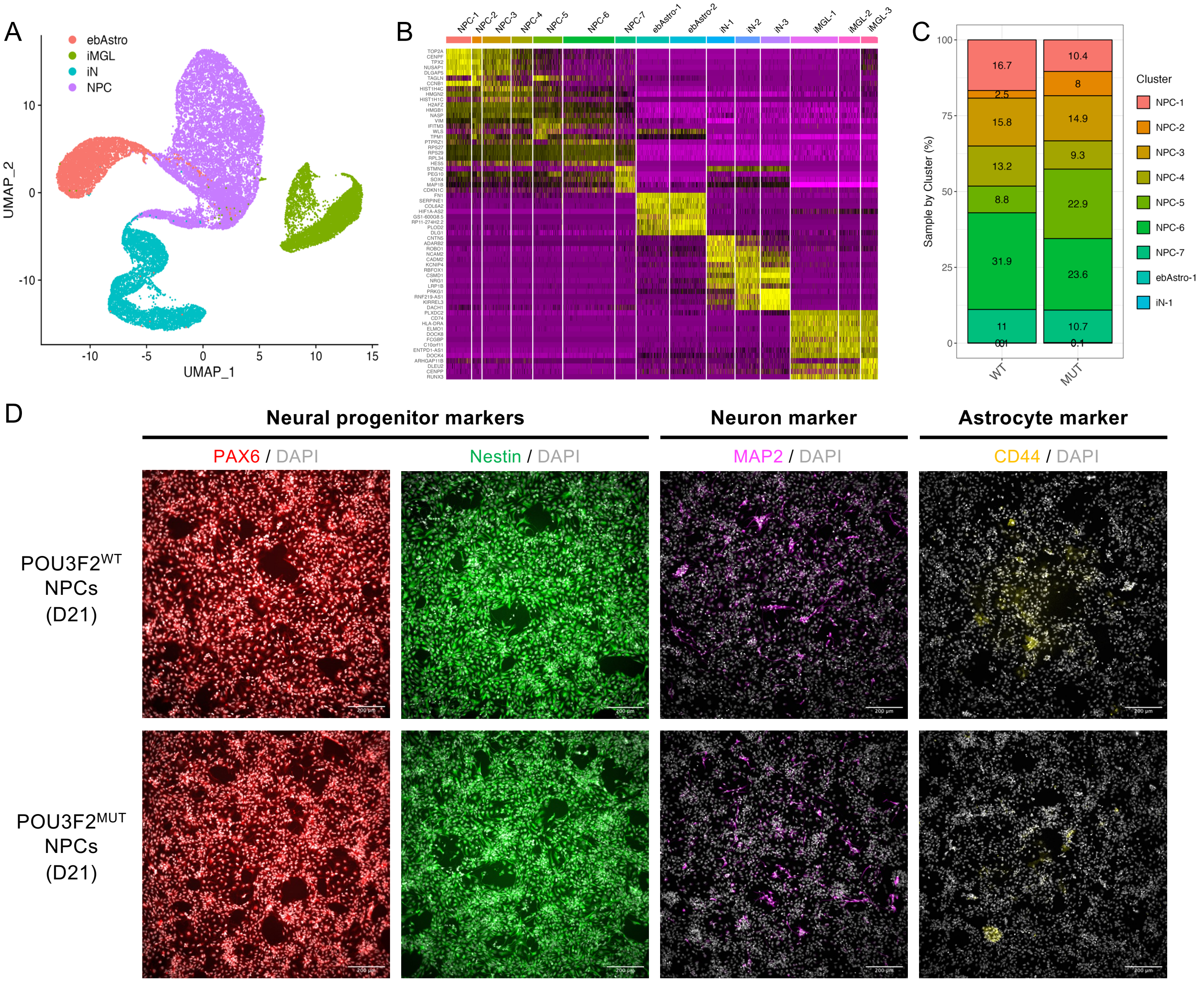

E

Line ID:	BR33		BR24		YZ1	
POU3F2 Status:	WT	MUT	WT	MUT	WT	MUT
% PAX6+/DAPI	82 ± 5%	82 ± 4%	76 ± 2%	79 ± 3%	75 ± 2%	66 ± 3%
% Nestin+/DAPI	93 ± 2%	92 ± 2%	89 ± 1%	91 ± 2%	87 ± 1%	85 ± 2%
% MAP2+/DAPI	14 ± 3%	14 ± 3%	15 ± 3%	17 ± 5%	12 ± 1%	9 ± 2%
% CD44+/DAPI	3 ± 1%	2 ± 1%	4 ± 1%	4 ± 1%	2 ± 1%	3 ± 1%

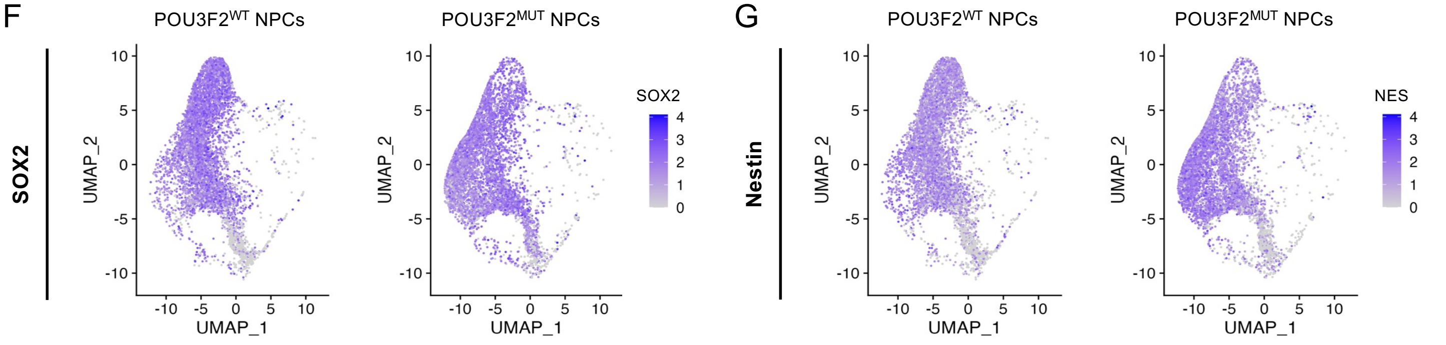

Supplementary Figure 2. POU3F2^{MUT} NPC cultures do not exhibit a shift in global cell-type identity. (A) UMAP of iPSC-derived brain cell types, with cells colored by sample identity. (B) Heatmap of the top 5 cluster markers per cluster in the single-cell analysis of iPSC-derived brain cell types. (C) Percent of cluster for NPCs, separated by *POU3F2* genotype. (D) Representative immunocytochemistry of PAX6 (NPC marker, red), Nestin (NPC marker, green), MAP2 (neuronal marker, magenta), and CD44 (astrocyte marker, yellow) in POU3F2^{WT} and POU3F2^{MUT} NPCs. Scale bar = 200 μm. (E) Quantification of the percentage of PAX6+/DAPI, Nestin+/DAPI, MAP2+/DAPI, and CD44+/DAPI cells in POU3F2^{WT} and POU3F2^{MUT} NPCs, mean ± SD (n = 9 fields per well, 1-2 wells per genotype). (F) UMAP of POU3F2^{WT} and POU3F2^{MUT} NPCs integrated with CS14 fetal brain data, colored by *SOX2* expression. (G) UMAP of POU3F2^{WT} and POU3F2^{MUT} NPCs integrated with CS14 fetal brain data, colored by *NES* expression.

Supplementary Figure 3

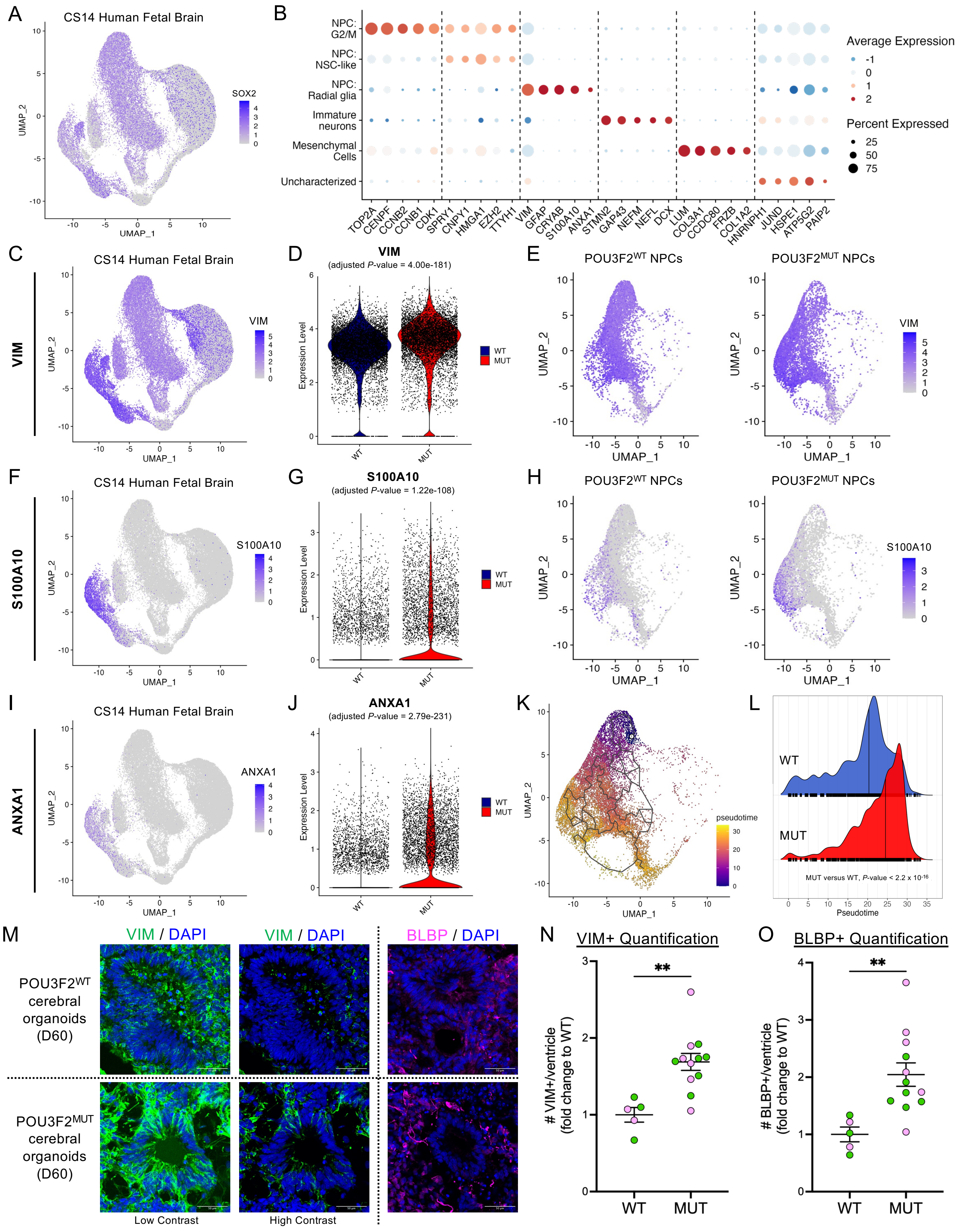

Supplementary Figure 3. POU3F2^{MUT} NPCs and cerebral organoids show enhanced radial glial identity. (A) UMAP of CS14 fetal brain single-cell data, colored by *SOX2* expression. (B) Scaled expression of markers used to define clusters in CS14 fetal brain single-cell data. (C) UMAP of CS14 fetal brain single-cell data, colored by *VIM* expression. (D) Violin plot of *VIM* expression in NPCs, stratified by *POU3F2* genotype. (E) UMAP of POU3F2^{WT} and POU3F2^{MUT} NPCs integrated with CS14 fetal brain data, colored by *VIM* expression. (F) UMAP of CS14 fetal brain single-cell data, colored by *S100A10* expression. (G) Violin plot of *S100A10* expression in NPCs, stratified by *POU3F2* genotype. (H) UMAP of POU3F2^{WT} and POU3F2^{MUT} NPCs integrated with CS14 fetal brain data, colored by *S100A10* expression. (I) UMAP of CS14 fetal brain single-cell data, colored by *ANXA1* expression. (J) Violin plot of *ANXA1* expression in NPCs, stratified by *POU3F2* genotype. (K) Pseudotime analysis of POU3F2^{WT} and POU3F2^{MUT} NPCs. (L) Ridge plot of pseudotime values, separated by genotype. (M) Immunohistochemistry of VIM (radial glia marker, green) or BLBP (radial glia marker, magenta) in POU3F2^{WT} and POU3F2^{MUT} cerebral organoids. Scale bar = 50 μm. (N-O) Quantification of the number of high-expressing (N) VIM+ or (O) BLBP+ cells per ventricle in POU3F2^{WT} and POU3F2^{MUT} cerebral organoids, mean $\pm$ SEM (n = 5-12 organoids per genotype). Student's t-test, $^{**}P<0.01$.
