# Supplementary material for: POU3F2 regulates canonical Wnt signalling via SOX13 and ADNP to expand the neural progenitor population
Source: Brain. 2025 Jun 11;148(12):4325–44. doi: 10.1093/brain/awaf221 (PMC12677912; doi:10.1093/brain/awaf221)
Supplement: awaf221_Supplementary_Data [file awaf221_supplementary_data.zip › brain-2024-02937-File011.pdf]

Supplementary Figure 4

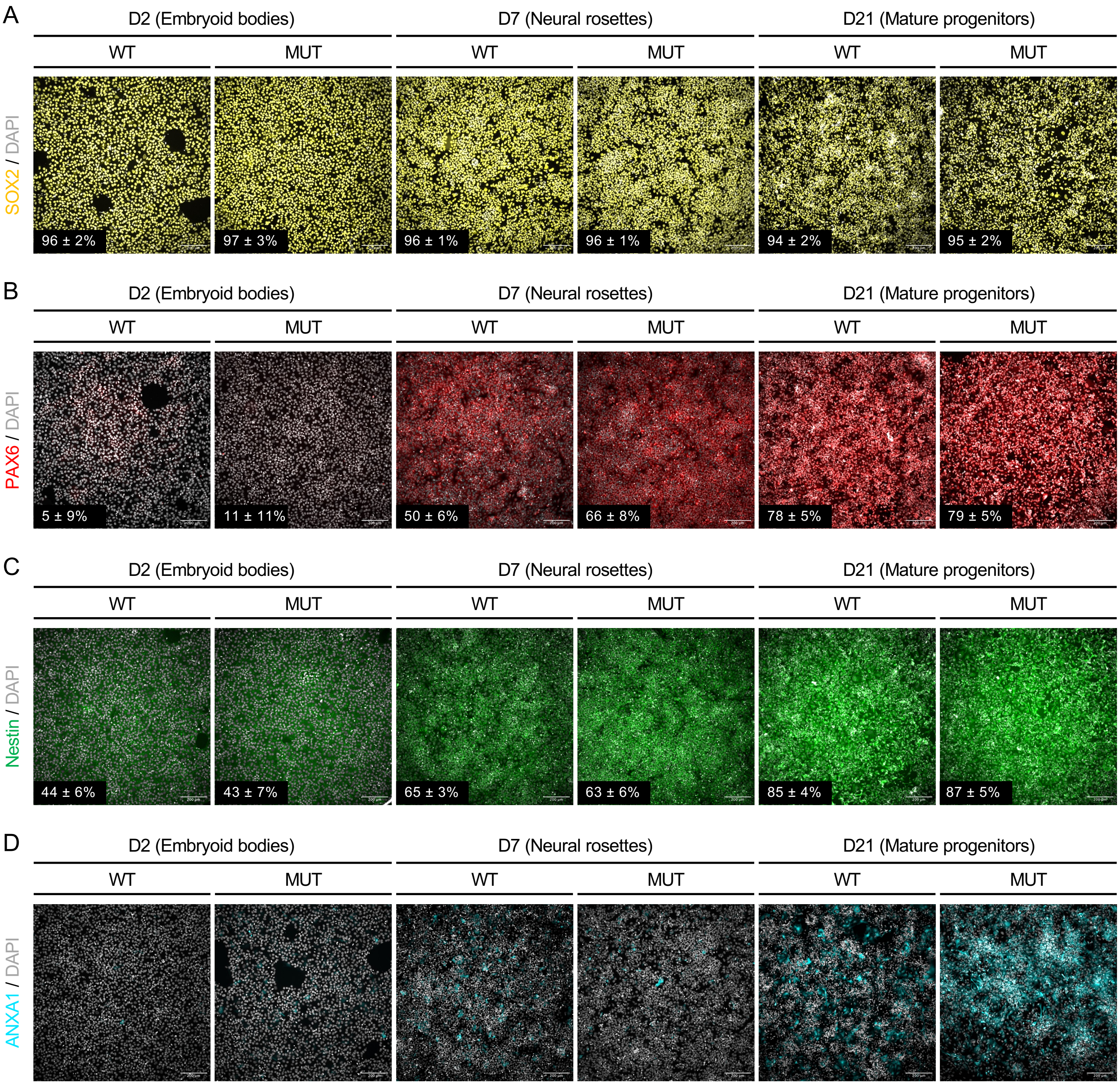

Supplementary Figure 4. Expansion of radial glia occurs after the onset of POU3F2 expression and changes in Wnt signalling. (A-D) Immunocytochemistry and quantification of (A) SOX2, (B) PAX6, (C) Nestin, and (D) ANXA1 (radial glia marker) in POU3F2^{WT} and POU3F2^{MUT} NPCs across time-course of dual-SMAD inhibition via embryoid body intermediate differentiation, mean ± SD (n = 9 fields per well, 2 wells per genotype). Scale bar = 200 μm.

Supplementary Figure 5

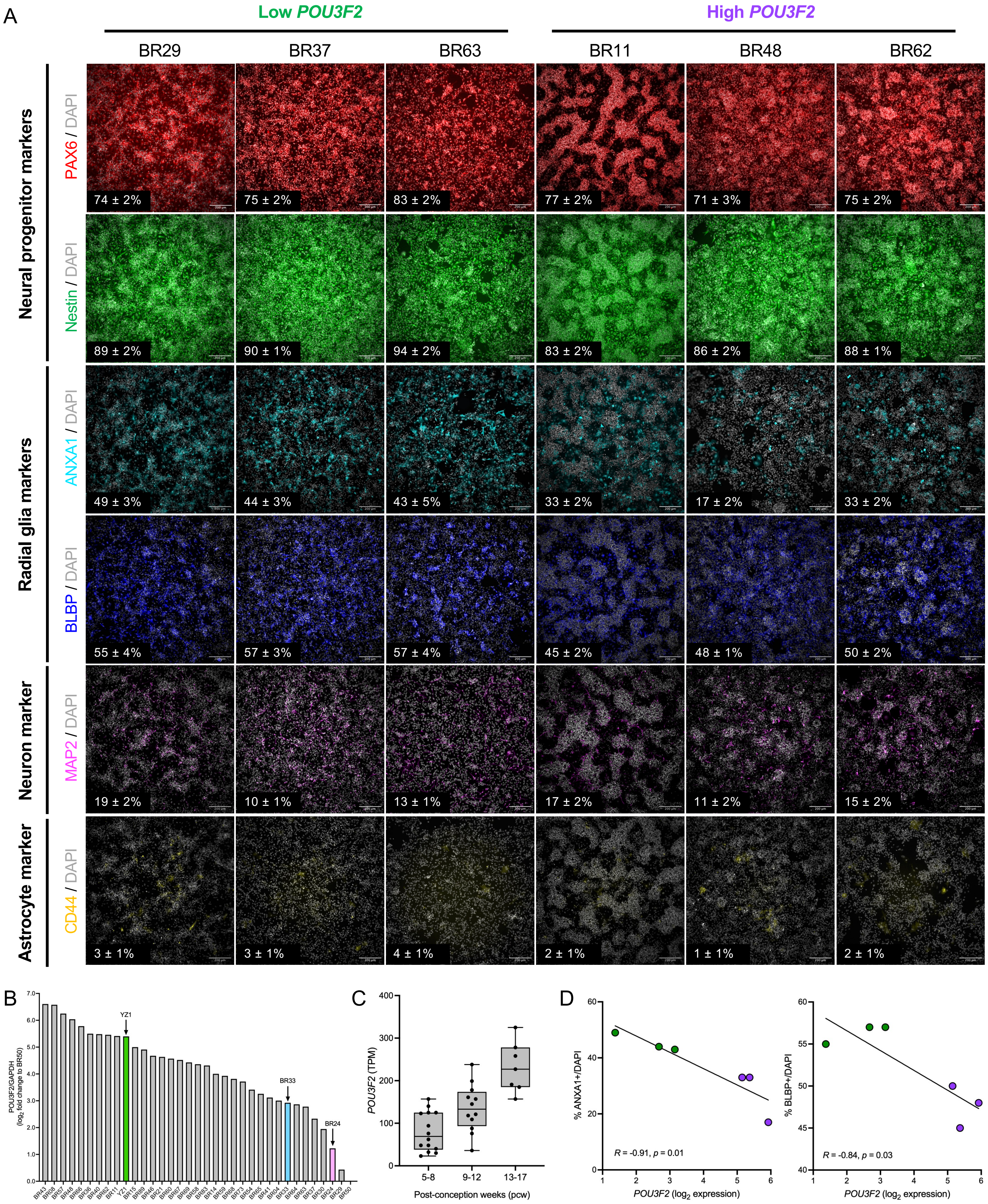

Supplementary Figure 5. *POU3F2* expression does not affect overall NPC differentiation but is anticorrelated with percent positivity of radial glia markers. (A) Immunocytochemistry and quantification of PAX6, Nestin, ANXA1, BLBP, MAP2, and CD44 in NPCs derived from ROSMAP participants with either high (BR11, BR48, BR62) or low (BR29, BR37, BR63) *POU3F2* expression, mean ± SD (n = 9 fields per well, 1 well per line). Scale bar = 200 μm. (B) Comparison of POU3F2 expression across ROSMAP cohort vs. the lines chosen for loss-of-function studies (YZ1/BR33/BR24), assayed by Western blotting and normalized to GAPDH. (C) Fetal brain transcriptomic data from the Human Developmental Biology Resource (HDBR) was obtained and filtered to only examine forebrain regions. Box and whiskers plots of *POU3F2* expression is shown, with each dot representing a single individual. (D) Association of *POU3F2* expression and ANXA1+/DAPI or BLBP+/DAPI percent positivity calculated in Supplementary Figure 5A. Pearson correlation coefficient and *P*-value shown.

Supplementary Figure 6

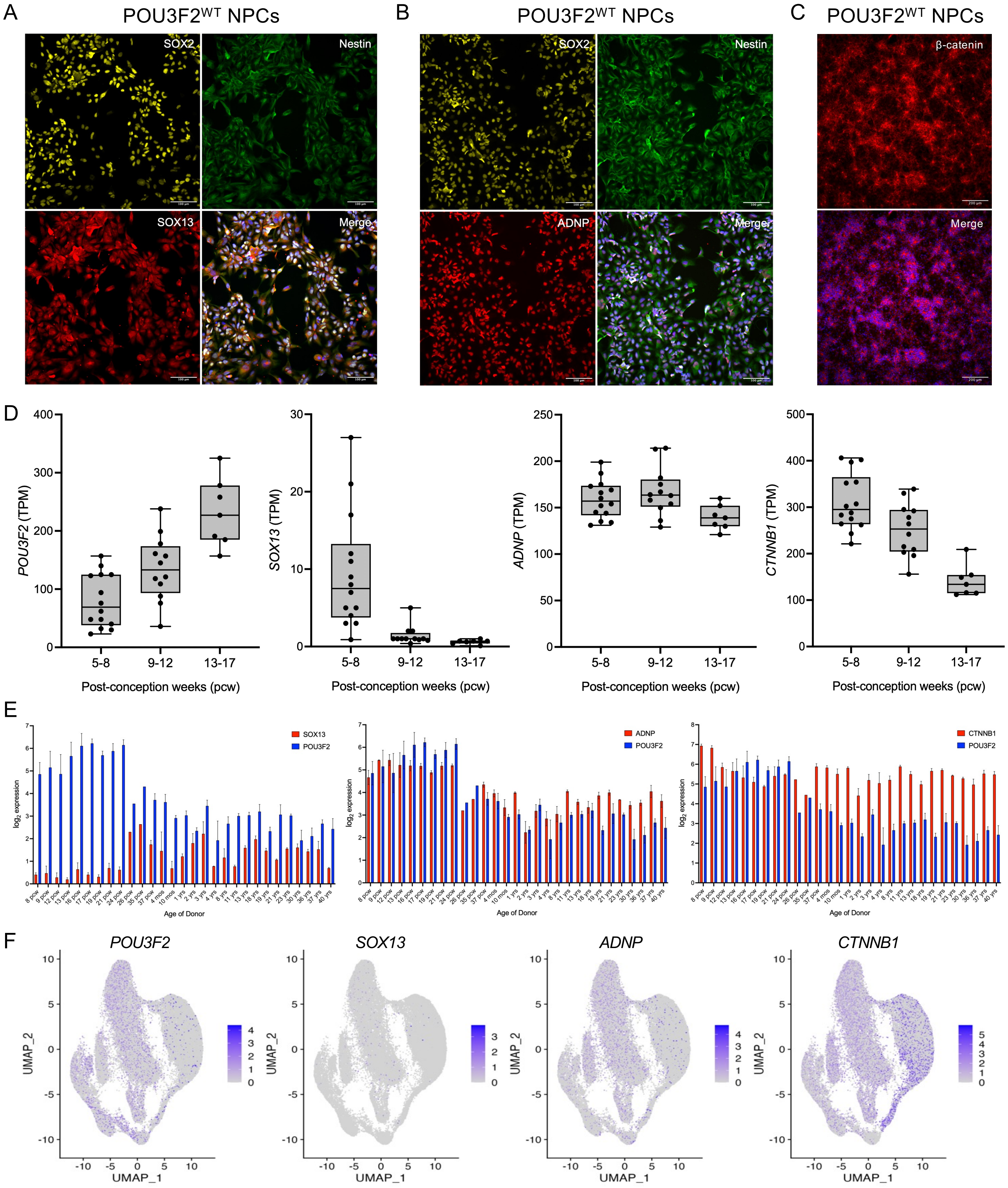

Supplementary Figure 6. SOX13 and ADNP are highly expressed in NPCs and at relevant timepoints during neurodevelopment. (A) Immunocytochemistry of SOX13 (red), SOX2 (yellow), and Nestin (green) in POU3F2^{WT} NPCs. Scale bar = 100 μm. (B) Immunocytochemistry of ADNP (red), SOX2 (yellow), and Nestin (green) in POU3F2^{WT} NPCs. Scale bar = 100 μm. (C) Immunocytochemistry of β-catenin (red) in POU3F2^{WT} NPCs. Scale bar = 200 μm. (D) Fetal brain transcriptomic data from the Human Developmental Biology Resource (HDBR) was obtained and filtered to only examine forebrain regions. Box and whiskers plots of expression of the following selected genes is shown, with each dot representing a single individual: *POU3F2*, *SOX13*, *ADNP*, *CTNNB1*. *POU3F2* expression is also shown in Supplementary Figure 5C. (E) Fetal brain transcriptomic data from the BrainSpan dataset was obtained to assess correlation of *POU3F2* expression with *SOX13, ADNP,* or *CTNNB1*. Average expression of selected genes across cortical regions (dorsal frontal, medial frontal, orbitofrontal, ventral frontal) for 1-3 individuals per developmental age is shown, mean ± SD. (F) UMAP of CS14 fetal brain data, colored by expression of selected genes of interest (*POU3F2, ADNP, SOX13, CTNNB1*).

Supplementary Figure 7

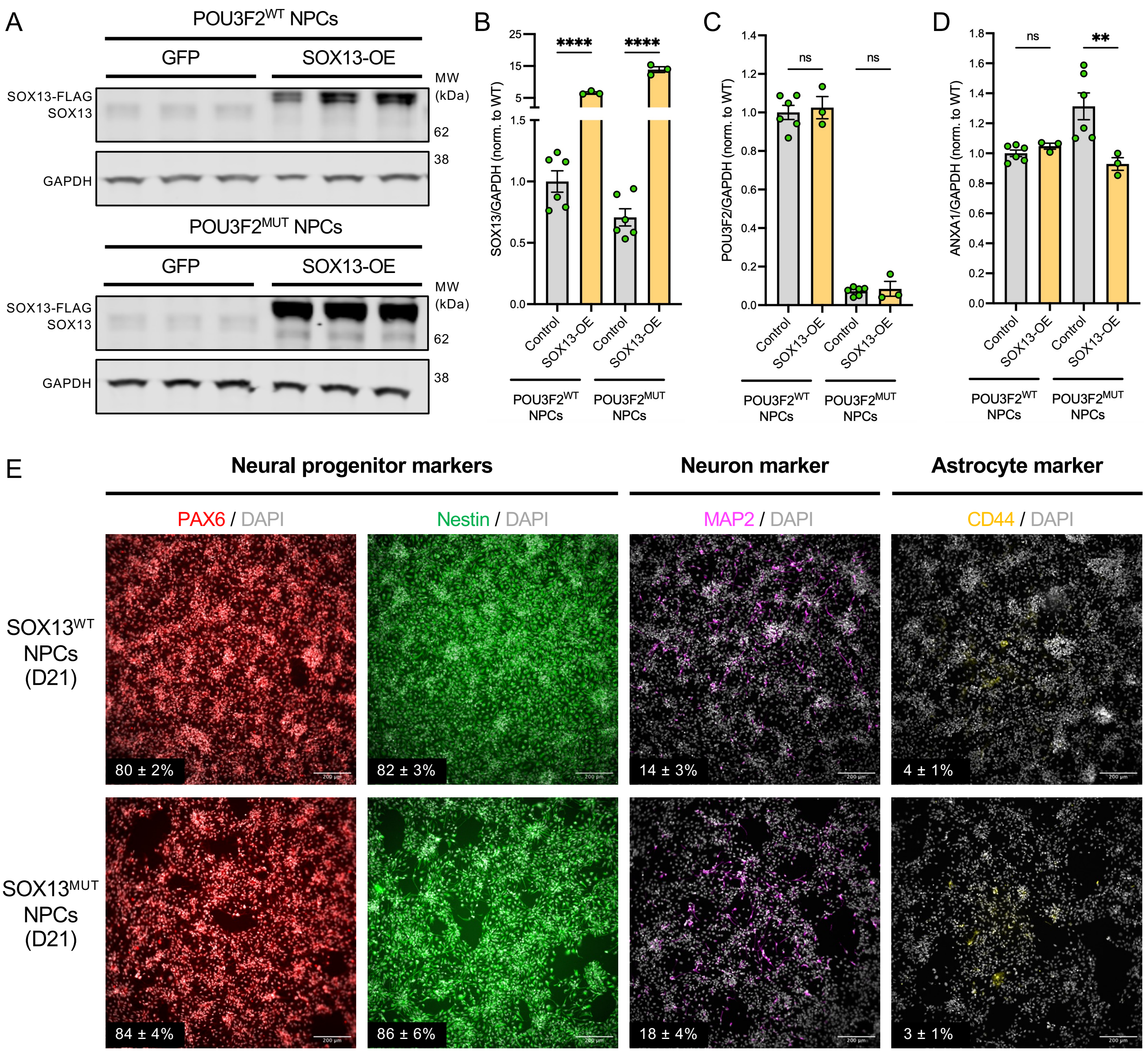

Supplementary Figure 7. SOX13 is a downstream transcriptional target of POU3F2 capable of Wnt modulation. (A) Western Blot analysis of POU3F2^{WT} and POU3F2^{MUT} NPCs overexpressing SOX13, probing for SOX13 and GAPDH. (B-D) Quantification of (B) SOX13, (C) POU3F2, or (D) ANXA1, normalized to GAPDH, mean ± SEM (n = 3-6 wells per category). ANOVA with post-hoc Sidak's test, ns = not significant, $^{**}P<0.01$, $^{****}P<0.0001$. (E) Immunocytochemistry and quantification of PAX6, Nestin, MAP2, and CD44 in SOX13^{WT} and SOX13^{MUT} NPCs, mean ± SD (n = 9 fields per well, 2-3 wells per genotype). Scale bar = 200 μm.
