# Supplementary material for: POU3F2 regulates canonical Wnt signalling via SOX13 and ADNP to expand the neural progenitor population
Source: Brain. 2025 Jun 11;148(12):4325–44. doi: 10.1093/brain/awaf221 (PMC12677912; doi:10.1093/brain/awaf221)

Full-length Western Blots (Figure 5)

Figure 5D

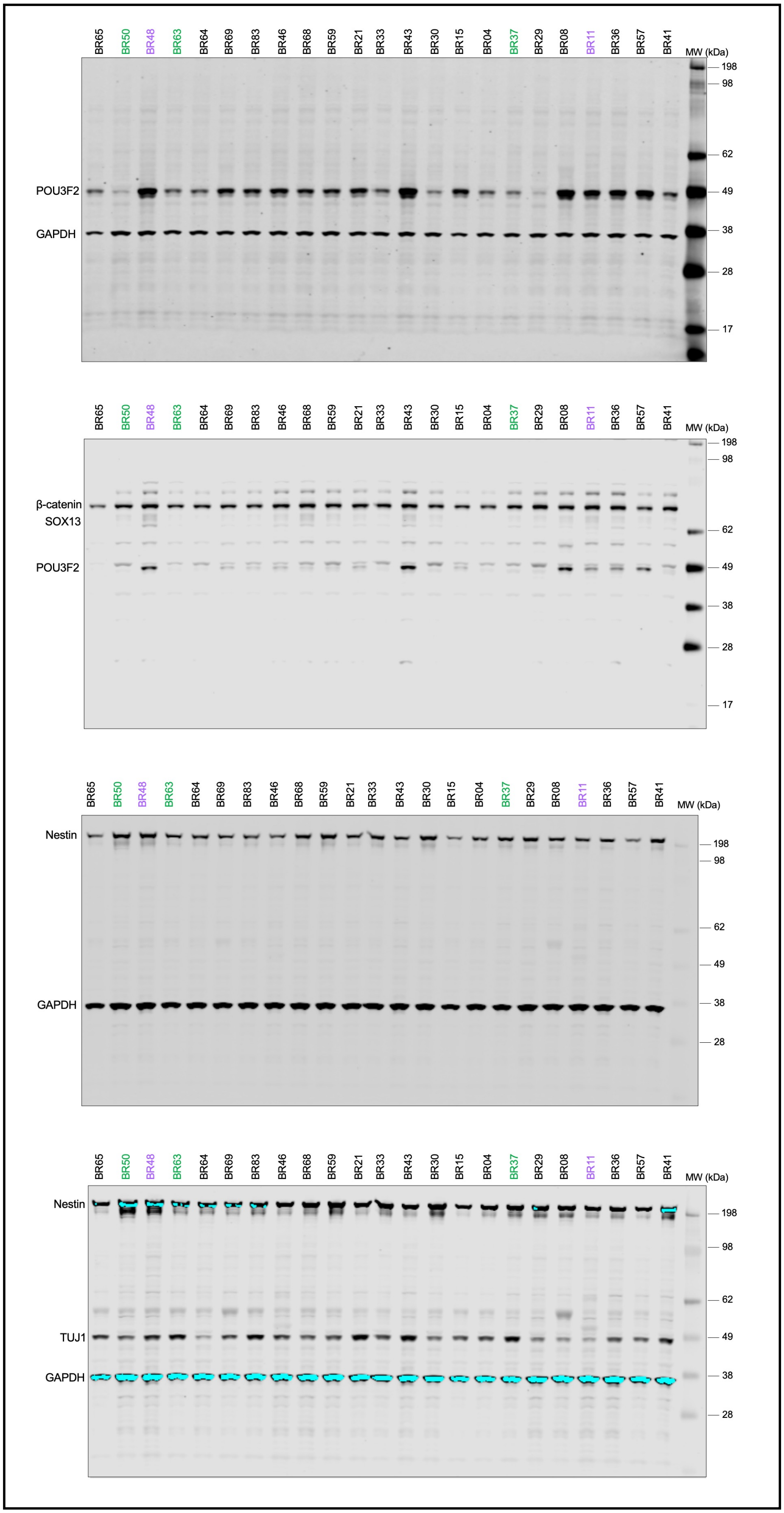

Full-length Western Blots (Figure 6)

Figure 6J

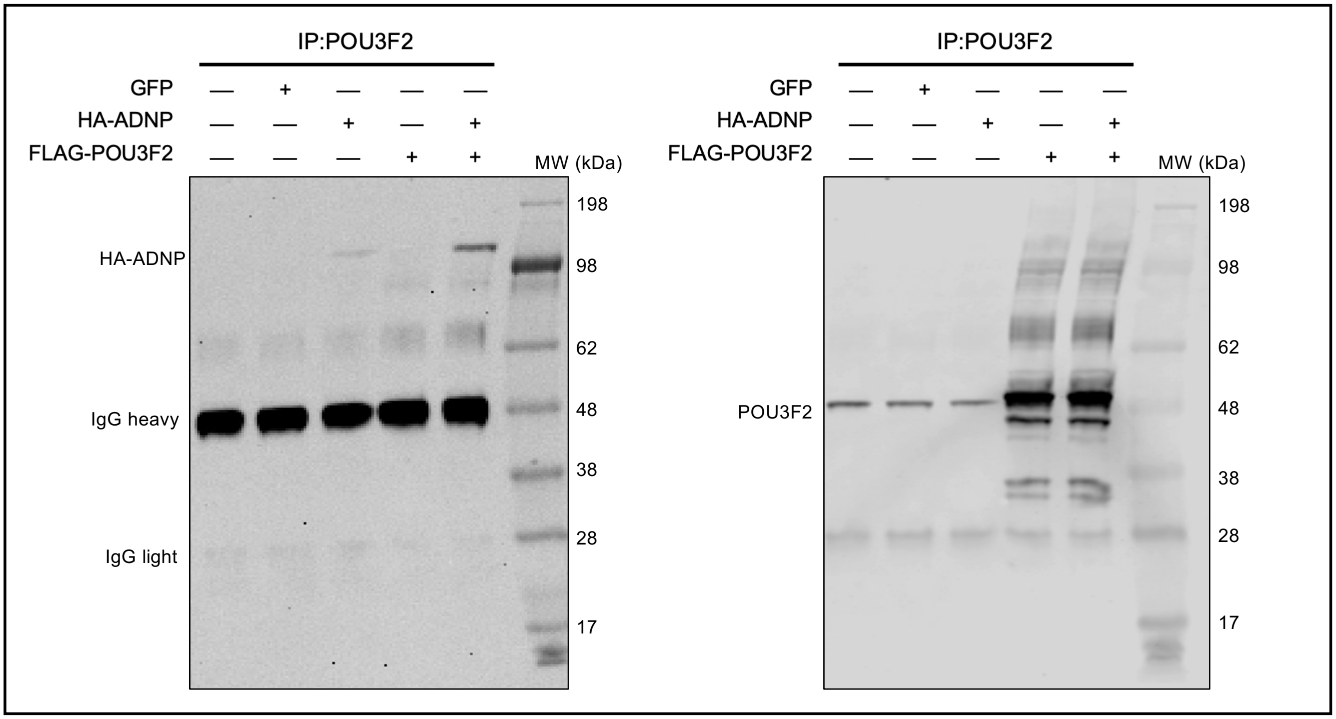

Full-length Western Blots (Figure 7)

Figure 7B

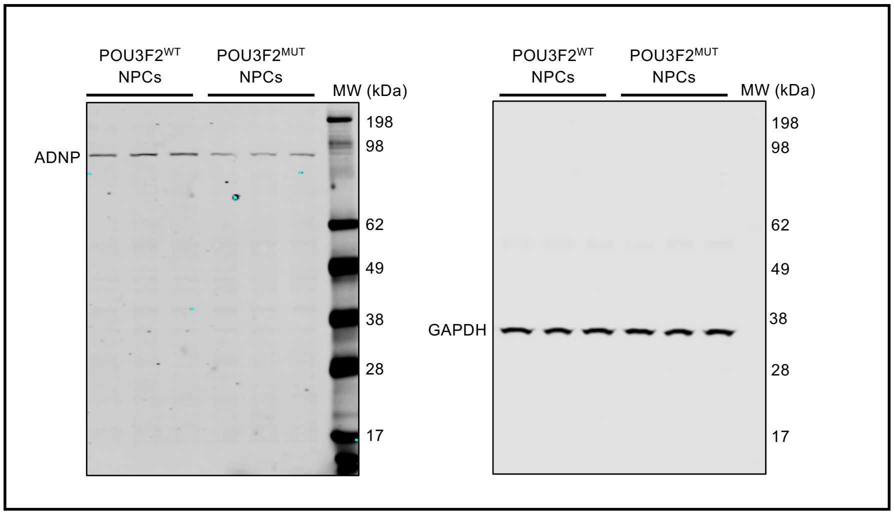

Figure 7C

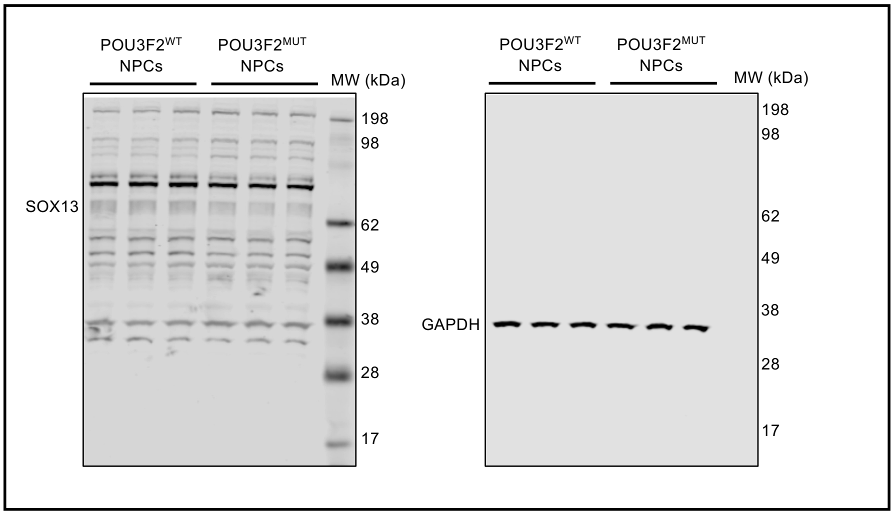

Figure 7I

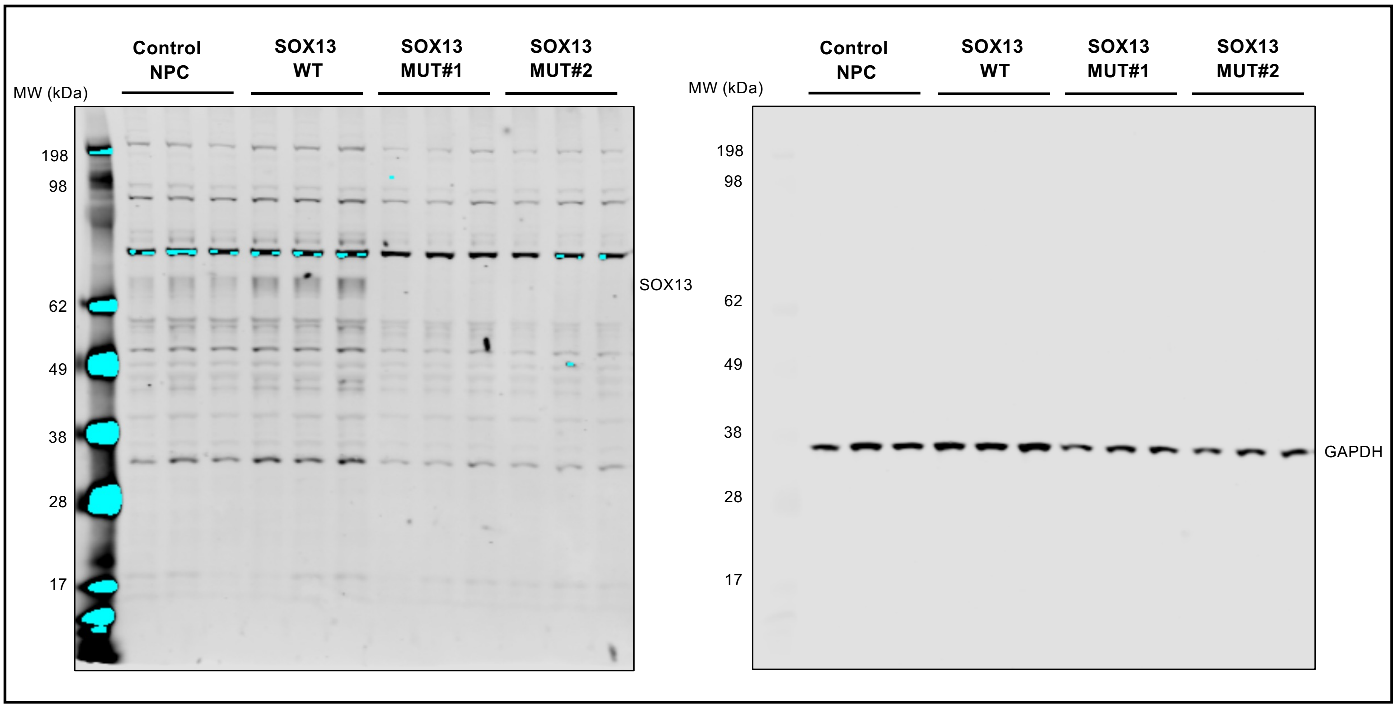

Full-length Western Blots (Supplementary Figure 1)

Supplementary Figure 1B

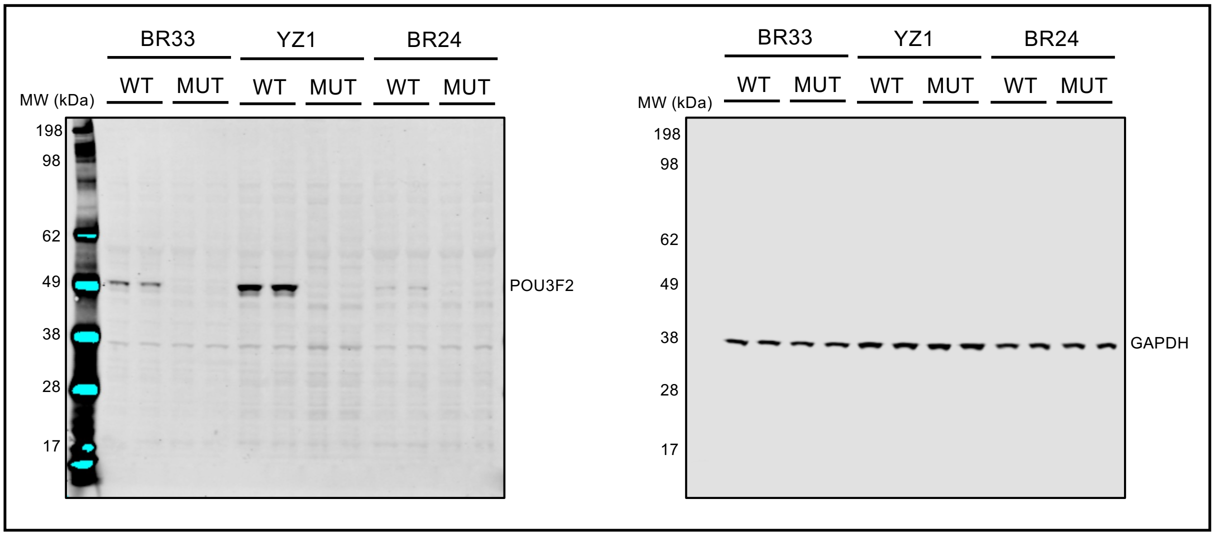

Full-length Western Blots (Supplementary Figure 7)

Supplementary Figure 7A

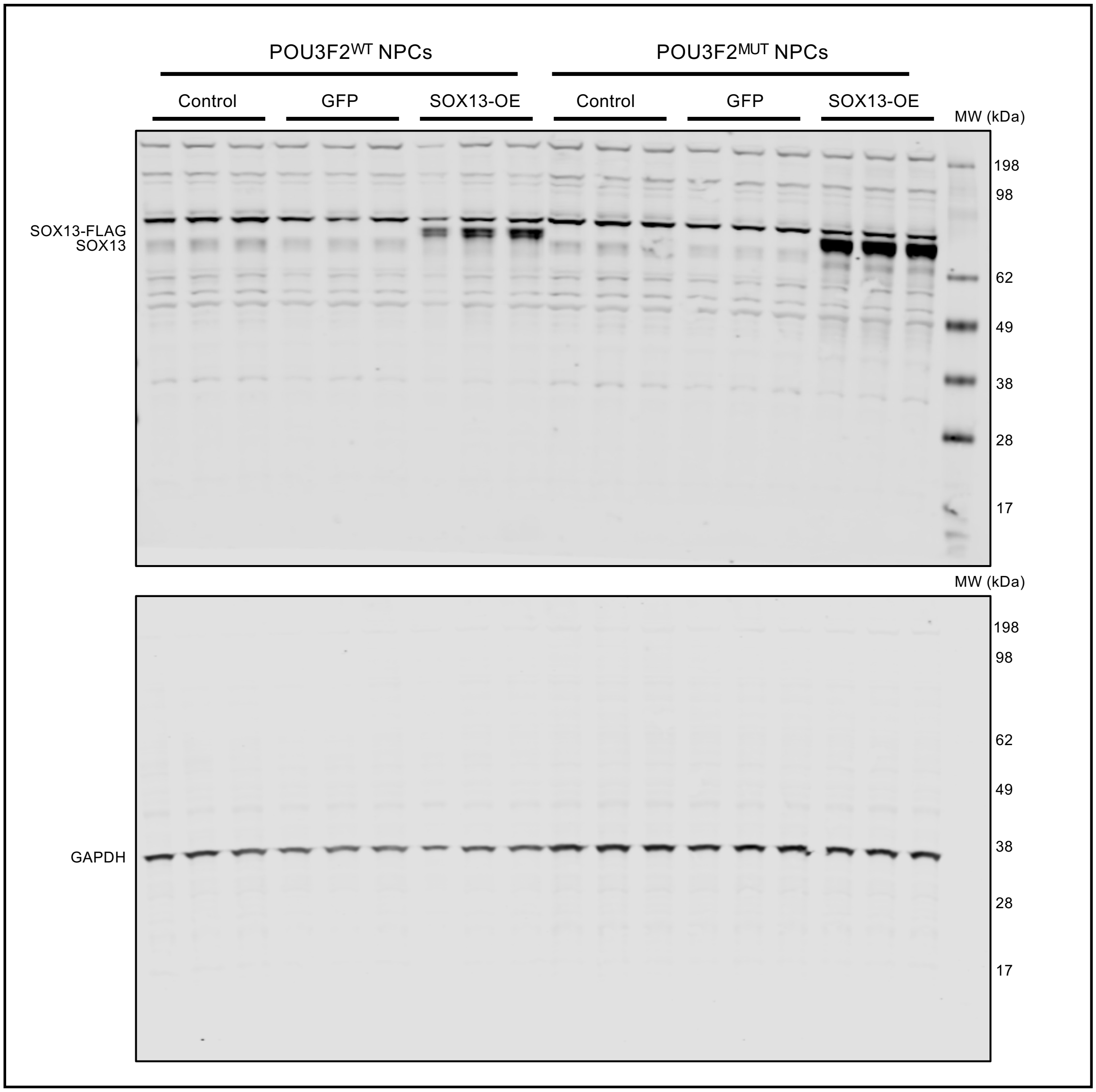

Supplement: awaf221_Supplementary_Data [file awaf221_supplementary_data.zip › brain-2024-02937-File017.pdf]
